# Degree of Fibrosis in Human Atrial Tissue Is Not the Hallmark Driving AF

**DOI:** 10.3390/cells11030427

**Published:** 2022-01-26

**Authors:** Kennedy S. Ramos, Lisa Pool, Mathijs S. van Schie, Leonoor F. J. M. Wijdeveld, Willemijn F. B. van der Does, Luciënne Baks, H. M. Danish Sultan, Stan W. van Wijk, Ad J. J. C. Bogers, Sander Verheule, Natasja M. S. de Groot, Bianca J. J. M. Brundel

**Affiliations:** 1 Department of Physiology, Amsterdam Cardiovascular Sciences, Amsterdam University Medical Centers, 1081 HV Amsterdam, The Netherlands; l.pool@erasmusmc.nl (L.P.); l.f.j.wijdeveld@amsterdamumc.nl (L.F.J.M.W.); Lucienne.baks@gmail.com (L.B.); h.m.d.sultan@amsterdamumc.nl (H.M.D.S.); s.w.vanwijk@amsterdamumc.nl (S.W.v.W.); 2 Department Cardiology, Erasmus Medical Center, 3015 GD Rotterdam, The Netherlands; m.vanschie@erasmusmc.nl (M.S.v.S.); w.vanderdoes@erasmusmc.nl (W.F.B.v.d.D.); n.m.s.degroot@erasmusmc.nl (N.M.S.d.G.); 3 Department of Cardiothoracic Surgery, Erasmus Medical Center, 3015 GD Rotterdam, The Netherlands; a.j.j.c.bogers@erasmusmc.nl; 4 Department of Physiology, University Maastricht, 6211 LK Maastricht, The Netherlands; s.verheule@maastrichtuniversity.nl

**Keywords:** atrial fibrillation, fibrosis, (bio)markers, cardiac mapping, structural remodeling, electrical remodeling

## Abstract

Background: The current paradigm is that fibrosis promotes electrophysiological disorders and drives atrial fibrillation (AF). In this current study, we investigated the relation between the degree of fibrosis in human atrial tissue samples of controls and patients in various stages of AF and the degree of electrophysiological abnormalities. Methods: The degree of fibrosis was measured in the atrial tissue and serum of patients in various stages of AF and the controls. Hereto, picrosirius and H&E staining were performed to quantify degree of total, endo-perimysial fibrosis, and cardiomyocyte diameter. Western blot quantified fibrosis markers: neural cell adhesion molecule, tissue inhibitor of metalloproteinase, lysyl oxidase, and α-smooth muscle actin. In serum, the ratio carboxyl-terminal telopeptide of collagen/matrix-metalloproteinase1 was determined. High-resolution epicardial mapping evaluated low-voltage areas and conduction abnormalities. Results: No significant differences were observed in the degree of fibrosis between the groups. Finally, no significant correlation—absolute nor spatial—was observed between all electrophysiological parameters and histological fibrosis markers. Conclusions: No differences in the degree of fibrosis were observed in patients from various stages of AF compared to the controls. Moreover, electrophysiological abnormalities did not correlate with any of the fibrosis markers. The findings indicate that fibrosis is not the hallmark of structural remodeling in AF.

## 1. Introduction

Atrial fibrillation (AF) is the most common progressive tachyarrhythmia in the Western world that is associated with the presence of an extensive arrhythmogenic substrate [1], which is called ‘electropathology’. Electropathology is defined as structural remodeling in atrial myocardium, which causes electrical conduction impairment. Electrophysiological studies revealed that cell to cell electrical conduction impairment includes conduction block and delay and epicardial breakthroughs, especially present in persistent AF (PerAF) patients [2] when compared to patients with acutely induced AF [3]. Additionally, it has been suggested that low-voltage areas can be a surrogate marker for arrhythmogenicity, representing areas of slower conduction [4]. It has been hypothesized that altered extra-cellular matrix (ECM), especially fibrosis, is the key aspect of structural remodeling that underlies these electrical conduction and voltage changes [5]. Although a plausible hypothesis, thus far, consistent clinical evidence is not available.

Fibrosis is a common pathological feature described as excessive deposition of ECM proteins (mainly collagen) in the cardiac interstitial space that can be observed during aging of the heart, and almost all forms of heart diseases [6,7]. During cardiac stress, proliferation of cardiac fibroblasts and subsequent trans-differentiation into activated cardiac myofibroblasts occurs [8]. Cardiac myofibroblasts express contractile proteins including α-smooth muscle actin (αSMA) [9] and secrete excessive amounts of ECM, thereby promoting fibrosis [8]. Fibrotic status also influences the quality of the ECM network by decreasing the collagen modulation by matrix metalloproteinase 1 (MMP1) and increasing its endogenous tissue inhibitor factor (TIMP) levels [10,11] as well as increasing the ECM crosslinking protein lysyl oxidase (LOX) activity and expression, resulting in higher resistance against degradation [12,13]. Additionally, neural cell adhesion molecule (NCAM), a marker for satellite cells and used to detect hepatic fibrosis, is expressed in the heart [14].

Importantly, distribution of interstitial fibrosis seems to influence the cardiomyocyte function differently. Excessive deposition of ECM separating individual cardiomyocytes, defined as endomysial fibrosis, results in altered electrical impulse propagation [15]. Additionally, increased deposition of ECM surrounding cardiomyocyte bundles, defined as perimysial fibrosis, may contribute to myocardial stiffness once these fibers are capable of storing kinetics into elastic energy and, as such, promote tensile strength [16].

Clinical evaluation of fibrosis in AF patients still faces difficulties. Although cardiac magnetic resonance imaging is often used to assess fibrosis non-invasively, there is still a lack of consensus on the utilized protocols [17]. In addition to the limited availability of human atrial tissue, studies have shown contradicting findings with respect to the degree of fibrosis in atrial tissue. Whereas some studies have revealed elevated levels of fibrosis between AF and control patients in sinus rhythm [18], others have reported no difference in fibrosis between these groups [19]. Moreover, data correlating the degree of fibrosis with the amount of electrical conduction impairment and low voltage throughout the different stages of AF is lacking.

The aim of this study was to evaluate whether fibrosis represents a key hallmark of electropathology in patients with AF. Hereto, we investigated the association between the degree of fibrosis with (1) AF stage in atrial appendage tissue and serum samples of patients with paroxysmal (ParAF), persistent (PerAF) and long-standing persistent AF (LSPerAF), and (2) the amount of electrical conduction impairment and low-voltage areas. The findings of the current study elucidate that the degree of fibrosis was comparable in the right and left atrial appendages (RAA; and LAA) of patients with ParAF, PerAF, or LSPerAF and controls in sinus rhythm (SR) without history of AF. Furthermore, in our study, the degree of fibrosis did not correlate with the amount of electrical conduction impairment nor with the presence of atrial low-voltage areas. This knowledge indicates that fibrosis is not the unique root cause of electropathology and AF promotion.

## 2. Materials and Methods

### 2.1. Study Population

The study population consisted of a group of 115 patients with or without a history of AF, subdivided in ParAF (N = 21), PerAF (N = 28), LSPerAF (N = 17), and a control group without history of AF (N = 49) undergoing cardiac surgery due to coronary artery disease and/or valvular heart disease or correction of congenital heart defect. All patients were enrolled from the HALT & REVERSE trial (MEC-2014-393) [20] at the department of Cardiology and Cardiothoracic Surgery in the Erasmus Medical Center, Rotterdam, The Netherlands. All patients signed written informed consent prior to inclusion. The study was carried out according to the principles of the Declaration of Helsinki in accordance with the Medical Research Committee involving the Human Subjects Act.

### 2.2. Blood and Tissue Sampling

Before the procedure, blood samples were collected in BD Vacutainer™ SST™ II Advance Tubes (Fisher Scientific, Breda, The Netherlands), and its serum obtained by centrifugation at 2000× *g* for 10 min at 4 °C and subsequently frozen in −80 °C until analysis. RAA was obtained from all patients (both with and without AF) from the incision used for extracorporeal circulation cannulation. Furthermore, amputation of the LAA was performed after cardioplegia in a selection of patients. Both RAA and LAA were immediately frozen in liquid nitrogen and stored at −80 °C. Due to limited tissue availability, not all patient samples could be utilized to analyze the various fibrosis endpoints.

### 2.3. Mapping Procedure

Epicardial high-resolution mapping was performed during open-heart surgery prior to commencement of extracorporeal circulation, as previously described in detail [21,22,23,24]. In short, a pacemaker wire temporarily attached to the right atrium (RA) free wall served as a bipolar reference electrode and a steel wire fixed to the subcutaneous tissue was used as an indifferent electrode [25]. Only during SR was epicardial mapping performed using unipolar arrays containing either 128 or 192 unipolar-electrodes, respectively, 0.65 and 0.45 mm of electrode diameter, and inter-electrode distances of 2 mm. From 35 patients, high resolution mapping in SR could be performed. When patients were in AF, electrical cardioversion was performed to convert them into SR. The mapping procedure was conducted by placing the electrode onto the RA, perpendicularly to the caval veins, on the RAA area as well as onto the LAA following the predefined mapping scheme [20,25]. At each mapping site, five seconds of SR were recorded including unipolar epicardial electrograms, a surface electrocardiogram (lead I), a bipolar reference electrogram, and a calibration signal (amplitude: 2 mV, duration: 1000 ms). At the LAA, the epicardial area mapped was marked exactly with stitches immediately before LAA amputation. Recordings were sampled at a rate of 1 kHz, amplified (gain: 1000), filtered (bandwidth: 0.5–400 Hz), analogue-to-digital-converted (16-bits), and stored on a hard disk.

### 2.4. Tissue Analysis for Western Blot and Immunohistochemistry

Part of the frozen RAA was cut into small fragments on dry ice and mixed with ice cold sample buffer (15% glycerol; 1% SDS; 12.5% 0.5 M Tris, pH 6.8; 2% bromophenol-blue solution and protease and phosphatase inhibitors). The tissue sample was homogenized using metal beads in the Qiagen Tissuelyser II for 3 min at 30 Hz, left on ice for 30 min for continued cell lysis and homogenized again for 3 min at 30 Hz. The lysates were centrifuged (20 min, 14.000 rpm at 4 °C), the supernatant was collected, passed through an insulin syringe, heated for 5 min at 95 °C, and samples were stored at −20 °C until analysis.

For western blot analysis, equal amounts (10 µg) of protein were separated on 4–20% Criterion TGX precast gels (Bio-Rad, Lunteren, The Netherlands) and transferred to nitrocellulose membranes (Bio-Rad). Membranes were blocked in 5% skim milk in TBST for 1 h at room temperature. Membranes were incubated overnight at 4 °C with the following primary antibodies in 3% BSA in TBST: anti-LOX (Abcam, #ab174316, Cambridge, UK), anti-NCAM (Abcam, #AF2408), anti-TIMP-1 (SantaCruz #SC5538, Santa Cruz, CA, USA), and anti-αSMA (Dako, #M0851, Carpinteria, CA, USA). Subsequently, these were incubated with secondary antibody (in 3% BSA in TBST) for 1 h at room temperature with horseradish peroxidase-conjugated goat-anti-rabbit or goat-anti-mouse antibodies (Dako Cytomation, Næstved, Denmark) depending on the species origin of the primary antibody. Signals were detected by the Amersham ECL prime western blotting detection reagent (GE Healthcare Life Sciences, Hoevelaken, The Netherlands) utilizing the Amersham Imager 600 (GE Healthcare Life Sciences) and quantified by densitometry (ImageQuantTL, GE Healthcare Life Sciences). Western blot analysis was performed at least in duplicate. Protein amounts were expressed relative to GAPDH for whole protein lysates.

For histochemical analysis, cryosections (5 µm) were obtained from frozen LAA and RAA samples, then stained with picrosirius red or hematoxylin and eosin. To determine the amount of fibrosis, cryosections were dried for 30 min, fixed with 4% paraformaldehyde in 0.1 M PBS for 5 min, stained in 0.1% Sirius red for 60 min, differentiated in 0.01 N HCl, immersed in seriate dehydration solutions (95%, 95%, and absolute ethanol), then cleared in xylene. For the determination of cardiomyocyte size and endo-perimysial distance, cryosections were dried for 30 min, stained in hematoxylin for 3 min, washed 3× in water, stained in eosin for 3 min, immersed in a series of washes (70%, 95%, and absolute ethanol), then quickly immersed in xylene. All cryosections were mounted with non-aqueous DPX medium. Photographs were taken at 10× magnification to recognize the epicardium and endocardium; subsequently, six pictures at magnification 40× were taken from each patient (half from a distance less than 300 μm from the epicardium and half from a distance less than 300 μm from the endocardium). Cardiomyocyte diameter, endomysial distance—space in between individual cardiomyocytes—and perimysial distance—space in between in between bundle bundles of cardiomyocytes—were analyzed and quantified by histomorphometry Image J 1.48 software (US National Institute of Health, Bethesda, MD, USA).

### 2.5. Serum Analysis for ELISA

MMP1 and CITP levels in serum samples were determined by ELISA measurements. For both parameters, serum was diluted 6× in 1% BSA in PBS. Levels were measured in triplicate using ELISA kits for MMP1 and CITP (ab215083 and LS-F55969, respectively) according to the manufacturer’s instructions.

### 2.6. Analysis of Mapping Data

Color-coded local activation maps were constructed using dedicated mapping software, AnnotationTool, based on annotations of the steepest negative deflection if the amplitude exceeded the noise level in the channel with a probability of 99.95%, assuming a minimum threshold of 0.05 mV/ms. Atrial extra systolic, aberrant, and ventricular beats were excluded. The RAA mapping site was subdivided in two halves: inter-caval and free wall. The free wall half of the mapping was used to analyze patterns of activation at the epicardial area closest to the tissue sample collection site. The LAA mapping site was uniformized to the distal 16 rows.

Consistent with previous mapping studies [26], differences in conduction time (CT) between neighboring electrodes (adjacent right and lower) were calculated and considered as conduction delay (CD) or conduction block (CB) when differences in local activation time (ΔCT) were between 7 and 11 or ≥12 ms, respectively. Lines of CB and continuous CDCB (CDCB) were defined as uninterrupted series of, respectively, inter-electrode CD, CB, or a combination of CD and CB. The lengths of these lines were measured and analyzed as the median length of lines per patient as well as the length of the longest line per patient. The percentage of CD/CB per mapping location was calculated using the described formulas [27]: potential voltages were defined as the peak-to-peak amplitude of the steepest deflection of unipolar potentials and considered low-voltage when <1 mV [28]. Local activation time maps were used to estimate local conduction velocity (CV). Local CV was computed from local activation times using discrete velocity vectors as previously described [29].

### 2.7. Statistical Analysis

Results are expressed as the mean  ±  standard error of the mean or median (min-max). Biochemical analyses were performed at least in duplicate. All data were tested for Gaussian distribution. Individual group mean differences were evaluated with the two-tailed Student’s *t* test, Mann–Whitney test, and Chi-square test; and Yate’s correction was applied for continuous variables. Correlation was performed with the Spearman correlation test. To compare continuous variables with a skewed distribution, the Mann–Whitney U test was applied. Values of *p* were two-sided and considered significant if <0.05. SPSS version 20 (IBM Analytics, Armonk, NY, USA), R Statistical Software (R Studio, Inc., Boston, MA, USA; version 1.0.153) and GraphPad Prism version 8.0 (Graphpad Software Inc., San Diego, CA, USA) were used for all statistical evaluations.

## 3. Results

### 3.1. Patient Characteristics

The study population consisted of 115 patients (72.17% male, 68 ± 10.75 years) including a control group of 48 (41.7%) patients without AF and a study group of 67 patients with either ParAF (N = 22, 19.1%), PerAF (N = 28, 24.3%), and LSPerAF (N = 17, 14.8%). Table 1 outlines the characteristics of the entire study population.

The parameters of age, sex, body mass index (BMI), hypertension, dyslipidemia, diabetes mellitus, thyroid disease, and left ventricular function were similar among the control and AF groups. AF patients more often use statins, digoxin, and anti-arrhythmic drug class III (see Table 1). The type of underlying heart disease varied between the control and AF groups (*p* < 0.001). From 48 patients without a history of AF, 24 developed post-operative AF. No significant difference in the degree of fibrosis were observed between patients with and without post-operative AF (Supplementary Figure S5).

### 3.2. Total, Endomysial, and Perimysial Fibrosis Levels, and Cardiomyocyte Diameter between the Various Stages of AF

**Figure 1:**
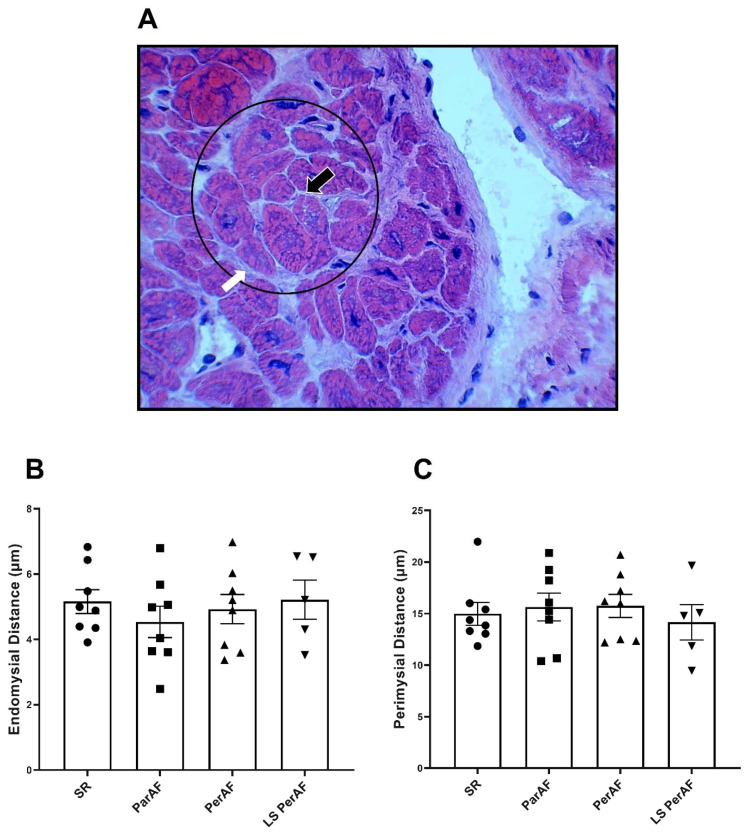
H&E staining reveals no increase in endo-perimysial fibrosis. In (**A**); representative example of cardiac bundle and perimysial space in human atrial tissue. Circle presents a cardiac bundle, white arrow shows perimysial space, and black arrow shows endomysial space. No significant difference between stages of AF and control SR group in terms of endomysial fibrosis (N = 8, SR; N = 8, ParAF; N = 8, PerAF; N = 5, LS PerAF) (**B**), and perimysial fibrosis (N = 8, SR; N = 8, ParAF; N = 8, PerAF; N = 5, LS PerAF) (**C**); respectively, *p* = 0.77 and *p* = 0.7542. SR = control group, ParAF = paroxysmal atrial fibrillation, PerAF = persistent atrial fibrillation, LSPerAF = longstanding persistent atrial fibrillation. Statistical tests used: ANOVA.

**Figure 2:**
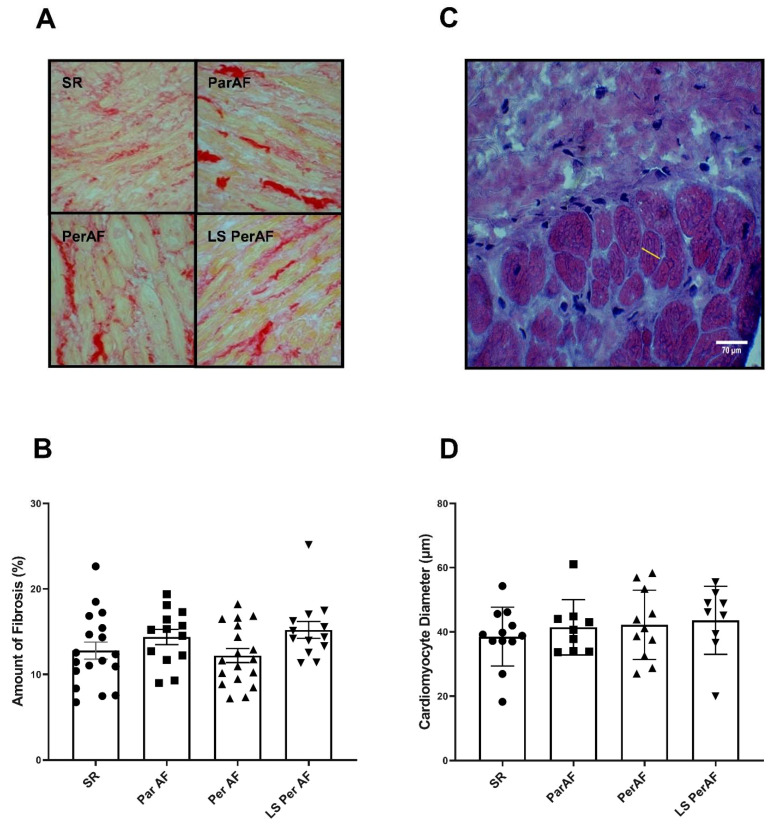
Picrosirius red and H&E stainings reveal no increase in fibrosis. No significant difference between various stages of AF and the control SR group in terms of the amount of fibrosis (N = 18, SR; N = 13, ParAF; N = 18, PerAF; and N = 13, LS PerAF) (**A**) and cardiomyocytel diameter (N = 12, SR; N = 9, ParAF; N = 11, PerAF; and N = 9, LS PerAF) (**B**); respectively, *p* = 0.12, *p* = 0.54. Picrosirius red staining in different stages of AF (**C**). In (**D**), yellow line shows the measurement of the cardiomyocyte diameter, H&E staining. SR = control group, ParAF = paroxysmal atrial fibrillation, PerAF = persistent atrial fibrillation, LSPerAF = longstanding persistent atrial fibrillation. Statistical tests used: ANOVA.

Hematoxylin and eosin (H&E) staining was performed in order to assess endomysial and perimysial fibrosis levels and cardiomyocyte size. No significant difference was present regarding endomysial distance in patients with ParAF (N = 8), PerAF (N = 8), LSPerAF (N = 5), and the control group (N = 8) (4.53 μm ± 1.36, 4.93 μm ± 1.27, 5.22 μm ± 1.34, and 5.16 μm ± 1.03, respectively, *p* = 0.77), as evident in Figure 1B. Similarly, perimysial distance did not vary significantly between patients with ParAF (N = 8), PerAF (N = 8), LSPerAF (N = 5), and the control group (N = 8) (15.64 μm ± 3.81, 15.74 μm ± 3.17, 14.16 μm ± 3.85, and 14.98 μm ± 3.12, respectively, *p* = 0.75), as seen in Figure 1C. In order to determine the total area of interstitial fibrosis in RAA, picrosirius red staining was performed. As shown in Figure 2B, the amount of total fibrotic (% of area) tissue was evaluated for the subgroups of AF and the control group, and we observed no significant difference between ParAF (N = 13), PerAF (N = 18), LSPerAF (N = 13), and the control groups (N = 18) (14.37 ± 3.22, 12.21 ± 3.47, 15.2 ± 3.55, 12.78 ± 4.24, respectively, *p* = 0.12). Furthermore, the level of cardiomyocyte hypertrophy was determined by measuring the diameter of atrial cardiomyocytes. Additionally, for this parameter, the diameter of atrial cardiomyocytes was comparable among ParAF (N = 9), PerAF (N = 12), LSPerAF (N = 8), and the control group (N = 12) (41.44 μm ± 8.59, 42.18 μm ± 10.75, 43.61 μm ± 10.6, 38.52 μm ± 9.149, respectively, *p* = 0.54), as depicted in Figure 2D. These findings show that there was no difference in the degree of interstitial fibrosis between ParAF, PerAF, LSPerAF, and the control group.

### 3.3. Determination of Additional Fibrosis Markers αSMA and TIMP in Various Stages of AF

**Figure 3:**
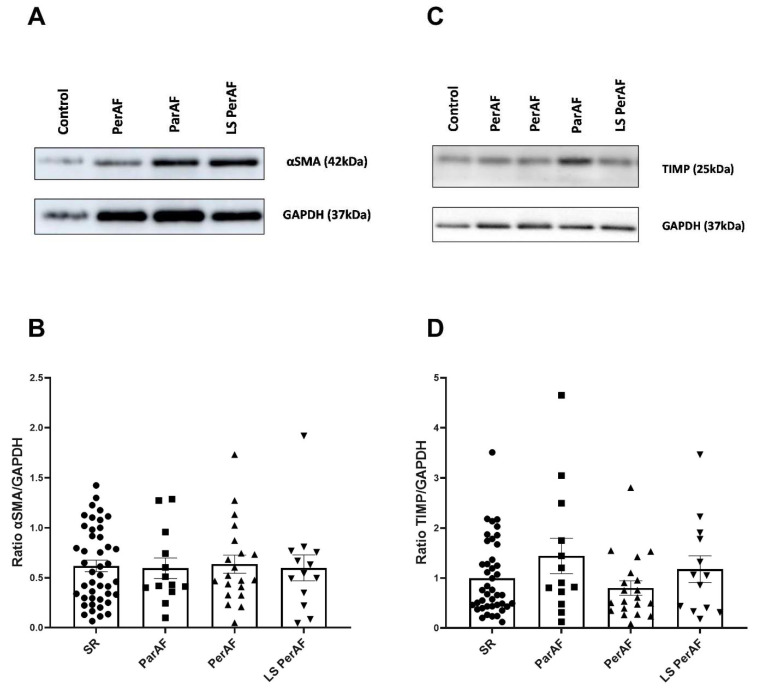
Cardiac myofibroblast (αSMA) marker and metalloproteinase inhibitor factor (TIMP). No significant difference was observed between various stages of AF and the control SR group regarding αSMA (N = 91) and TIMP (N = 89); respectively *p* = 0.99, (**B**), and *p* = 0.41, (**D**). Representative blot images for αSMA and TIMP expression are illustrated in (**A**,**C**), respectively. SR = control group, ParAF = paroxysmal atrial fibrillation, PerAF = persistent atrial fibrillation, LSPerAF = longstanding persistent atrial fibrillation. Statistical tests used: ANOVA.

αSMA is a marker for transdifferentiated cardiac myofibroblasts [30] and TIMP is a tissue inhibitor of metalloproteinases [10]. Their expressions were determined in RAA by western blot analyses. In accordance with all histological parameters, patients from ParAF, PerAF, LSPerAF, and the control group presented comparable αSMA levels (0.62 ± 0.38, 0.59 ± 0.37, 0.63 ± 0.41, 0.6 ± 0.47, respectively; *p* = 0.99), as shown in Figure 3B, and TIMP levels of 1.44 ± 1.28, 0.80 ± 0.64, 1.18 ± 0.96, 1 ± 0.74, respectively; *p* = 0.41), as seen in Figure 3D. All original blots are presented in the Supplementary Materials section, in Supplementary Figure S4. Thus, the levels of additional fibrosis markers αSMA and TIMP are comparable between ParAF, PerAF, LSPerAF, and the control group.

### 3.4. Determination of Additional Fibrosis Markers NCAM and LOX in Various Stages of AF

**Figure 4:**
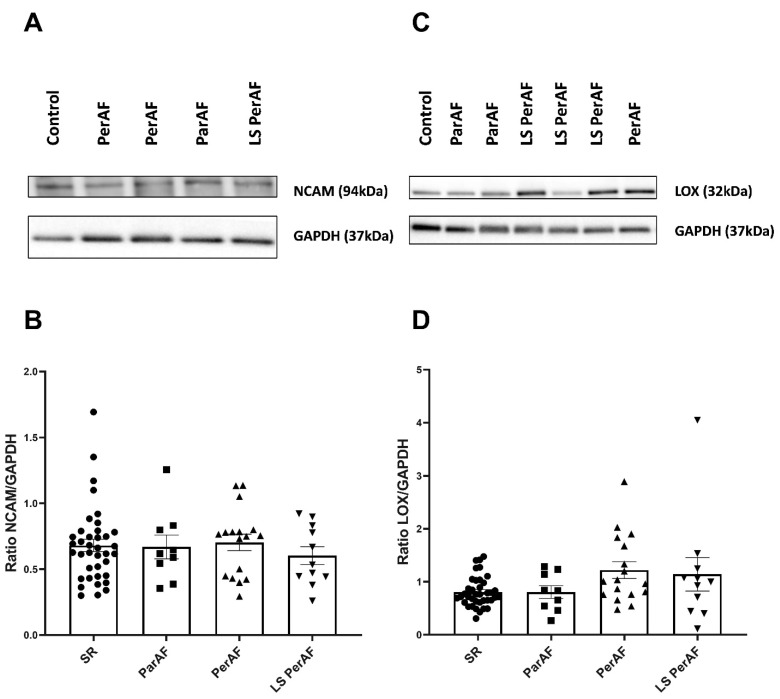
Fibroblast marker (NCAM and collagen crosslinking promoter (LOX). No significant difference was observed between various stages of AF and control SR group, in terms of NCAM (N = 76) (**B**) and LOX (N = 92) (**D**), respectively, *p* = 0.83 and *p* = 0.11. Representative blot images for NCAM and LOX expression are illustrated in (**A**,**C**), respectively. SR = control group, ParAF = paroxysmal atrial fibrillation, PerAF = persistent atrial fibrillation, LSPerAF = longstanding persistent atrial fibrillation. Statistical tests used: ANOVA.

By using western blot analyses, the levels of NCAM, a marker of satellite cells and (hepatic) fibrosis [14] and lysyl oxidase (LOX), an enzyme responsible for catalyzing the formation of cross-links between ECM proteins [13], were measured. In RAA, the levels of NCAM and LOX, observed in the subgroups ParF, PerAF, and LSPerAF, did not differ from the control group (NCAM levels in ParAF:0.7 ± 0.27, PerAF: 0.70 ± 0.26, LSPerAF: 0.6 ± 0.2, and SR: 0.68 ± 0.29; *p* = 0.83; and LOX levels in ParAF:0.81 ± 0.36, PerAF: 1.22 ± 0.64, LSPerAF: 1.14 ± 1.04, and SR: 0.81 ± 0.28; *p* = 0.11), as illustrated in Figure 4B,D. All original blots are presented in Supplementary Materials, in Supplementary Figure S4. The findings indicate that markers related to the development of fibrosis are comparable between different stages of AF and the control group.

### 3.5. Serum Fibrosis Marker: Ratio CITP:MMP1

**Figure 5:**
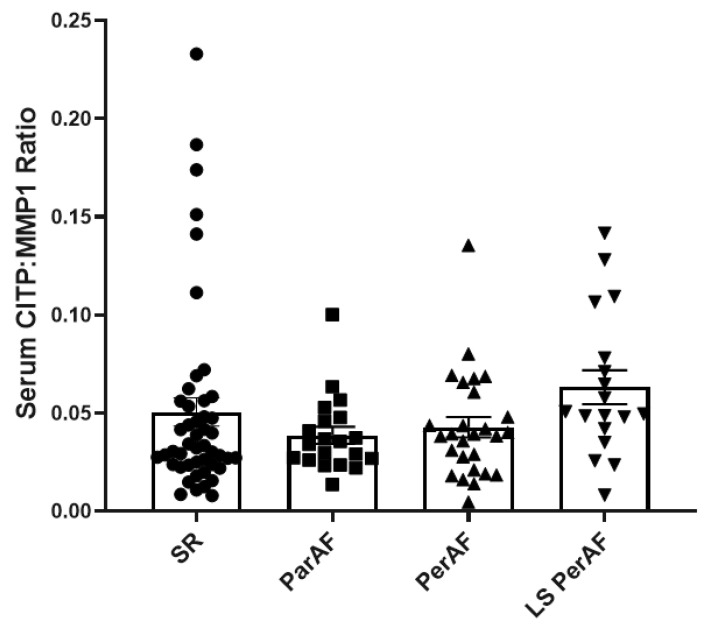
Blood based quantification of ratio CITP/MMP1. Ratio between the sub product of collagen degradation (CITP) and its promoter (MMP1) was assessed (N = 20, ParAF; N = 27, PerAF; N = 18, LS PerAF; and N = 47, SR) in line with the previous endpoints comparable values were observed at different stages of the AF and control SR group, *p* = 0.07. SR = control group, ParAF = paroxysmal atrial fibrillation, PerAF = persistent atrial fibrillation, LSPerAF = longstanding persistent atrial fibrillation. Statistical tests used: ANOVA.

Given that metalloproteinase 1 (MMP1) [10] degrades the collagen network, resulting in collagen I carboxy terminal telopeptide (CITP) release in the blood stream, we quantified MMP1 and CITP values in serum using the ELISA technique. As an assessment of collagen turnover, ratios CITP/MMP1 did not present significant difference between stages of AF and the control group (ParAF:0.04 ± 0.02, PerAF: 0.043 ± 0.03, LS PerAF: 0.06 ± 0.04, and control: 0.05 ± 0.05; *p* = 0.07), as illustrated in Figure 5. These findings indicate no increase in fibrosis markers in the sera of patients at different stages of AF compared to the controls. In addition, multivariate analyses showed no effect of clinical parameters such as diabetes, hypertension, BMI, and underlying cardiac diseases against any endpoint for the degree of fibrosis.

### 3.6. Correlation between Histological Fibrosis Markers and Electrophysiology

**Figure 6:**
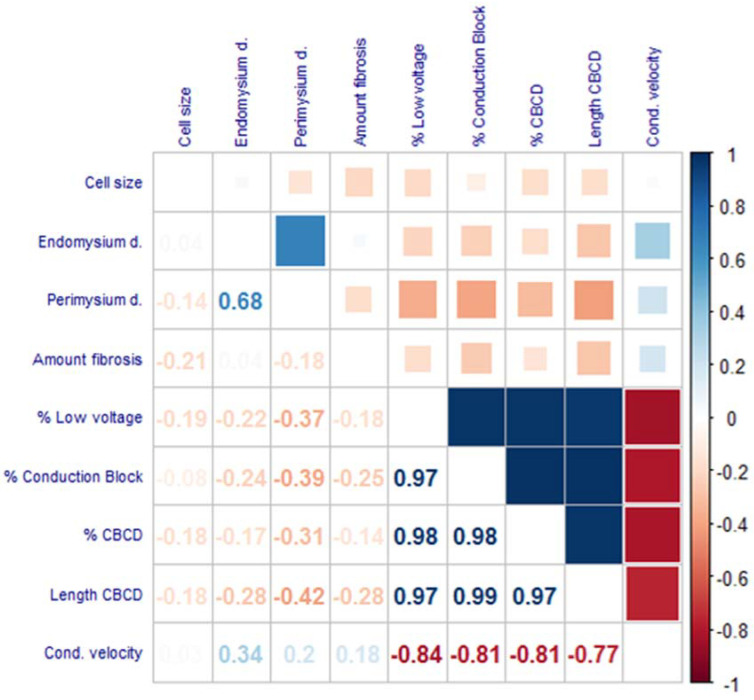
Correlogram with correlation coefficients for histological fibrosis markers and electrophysiological endpoints. No strong correlation (−0.8 < R < 0.8) was visualized between histological fibrosis markers and electrophysiological endpoints (N = 35). Statistical tests used: Spearman correlation test.

Generally, fibrosis is a culprit factor for impaired cardiac conduction as well as for areas characterized by low potential voltages [28]. Therefore, we correlated all previously mentioned histological fibrosis markers with electrophysiological parameters including percentage of CB, percentage of CBCD, length of CBCD lines, percentage of low voltage areas, and CV measured with epicardial mapping from the same location (i.e., RAA), in patients with or without a history of AF during sinus rhythm (Figure 6). Additionally, slower CV negatively correlated with a percentage of low-voltage areas, percentage of CB, and percentage of CBCD (R < −0.8). A negative trend was observed between CV and length of CBCD lines (R = −0.77).

After correlating the electrophysiological parameters with fibrosis markers, no significant correlations (−0.8 < R < 0.8, *p* > 0.05) were found between the histological fibrosis markers and any of the electrophysiological parameters measured during SR (Figure 6), suggesting that the association between conduction impairment and low-voltage areas with histologically quantified fibrosis was not present in this study population.

### 3.7. Spatial Analysis of Electrophysiological and Histological Staining

**Figure 7:**
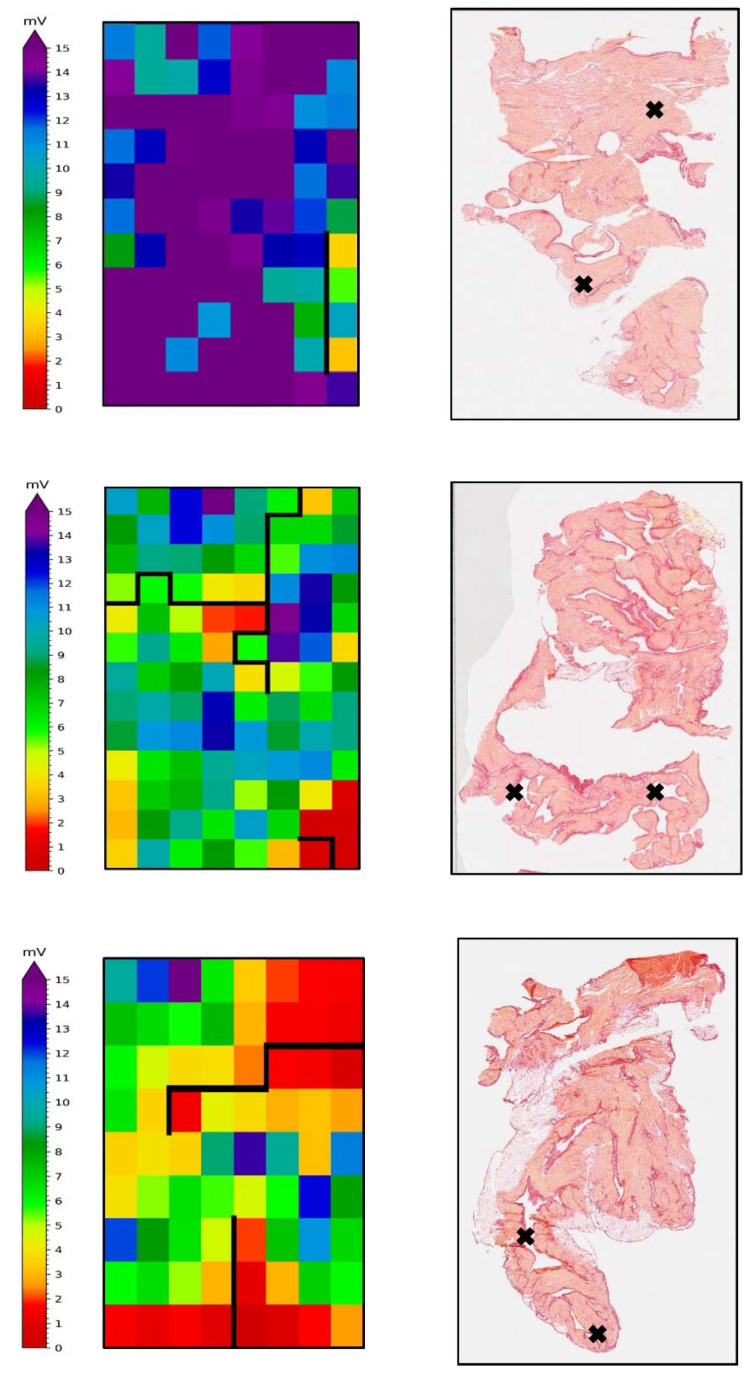
Spatial analysis of electrophysiological and fibrosis parameters in the LAA of patients with ParAF, PeAF, and LS PerAF. In the left panels, high resolution epicardial mappings reveal simultaneously color-coded degree of voltage, and black lines represent block and delay lines; on the right panels, correspondent LAA tissue was stained with picrosirius and percentage of total fibrosis was determined for each subdivision of the slide. Black crosses indicate sub-sectional areas presenting the highest amount of fibrosis.

In order to evaluate simultaneous fibrotic and electrophysiological status within the exact same epicardial area, mapped LAA were excised, sectioned, and stained with picrosirius red in order to assess the total amount of interstitial fibrosis. In comparison to the total stained tissue, areas containing higher percentages of total fibrosis did not correspond to areas with lower potential voltages, nor CB and CBCD lines (Figure 7), suggesting that epicardial electrophysiological abnormalities did not correspond to the histological assessment of fibrosis.

## 4. Discussion

In the current study, we observed in human atrial appendage tissue samples that histological fibrosis markers including cardiomyocyte size, total, but also endo and perimysial fibrosis, the tissue fibrosis markers αSMA, TIMP, NCAM, and LOX, and the serum fibrosis marker CITP/MMP1 ratio are comparable between various stages of AF and the control group. Therefore, these overall findings are in agreement and indicate no association between fibrosis levels and the investigated stages of clinical AF. Additionally, histological fibrosis markers did not correlate with electrophysiological parameters including conduction abnormalities and low voltage area, suggesting a lack of proportional relation between the degree of fibrosis and electrophysiological substrate in AF. Finally, in LAA, concomitant electrophysiological and histological analyses showed a lack of spatial association between local conduction abnormalities as well as low-atrial voltage areas in comparison with the total amount of fibrosis. Therefore, our findings do not support the paradigm that fibrosis is the unique culprit factor for electropathology driving AF.

### 4.1. Quantification of Biomolecular Markers for Atrial Fibrosis

To date, there is a great need for the detection of the AF substrate, in order to develop substrate-based therapies. Although atrial fibrosis is considered as an important substrate in AF pathogenesis, the detection of fibrosis in human atrial tissue samples remains difficult to perform. To our knowledge, this study is the first to evaluate, in a systematic fashion, nine different markers related to the development of fibrosis, using histochemistry, western blot, and ELISA techniques, and correlated the histological findings with electrophysiological parameters collected with high-resolution epicardial unipolar mappings of RAA and LAA from patients with and without a history of AF, during SR.

To determine the degree of fibrosis, our first approach was histological quantification of total, endomysial, and perimysial fibrosis, and cardiomyocyte hypertrophy in atrial appendage tissue samples. No differences between various stages of AF and controls without history of AF were observed. Similar to our findings, in an experimental goat model for chronic “lone” AF [31], no increase in the amount and distribution of fibrosis was observed after chronically sustained AF (20 weeks), suggesting that persistence of AF does not lead to higher degree of fibrosis. In contrast, experimental studies in dogs suggest preexisting fibrosis due to chronic heart failure as the culprit for the development of AF [7]. A clinical study using septal atrial tissue stained with H&E and Masson’s trichrome revealed that 25% of patients with a history of AF did not present an excessive amount of interstitial fibrosis, and approximately 15% displayed cardiomyocyte hypertrophy [32], suggesting that fibrosis features do not always accompany AF and instigates awareness to other concomitant pathological mechanisms underlying AF. In contrast, another clinical study, using post-mortem (causes of death: acute myocardial infarction, pulmonary embolism and stroke) tissue samples collected from different atrial sites including superior pulmonary veins, inferior pulmonary veins, center of posterior left atrial wall, crista terminalis, and Bachmann’s bundle revealed an increase in the percentage of fibrosis in patients with a history of AF in comparison with patients without a history of AF [33]. The findings did not show any difference in histological fibrosis markers among the five sampling locations, which is in conflict with their suggested hypothesis of a culprit relationship between fibrosis and AF progression [33] with another electrophysiological study that showed a prominent degree of conduction impairment in Bachmann’s bundle in comparison with other atrial sites [34]. Interestingly, the post-mortem study [33] also revealed that the absolute values of percentage of fibrosis in patients without a history of AF was lower in crista terminalis. When compared to what we observed in RAA (8 ± 5% post-mortem study and 12.78 ± 4.24% current study), the findings indicate that our control patients without a history of AF already presented considerable levels of atrial fibrosis. This fact is possibly related to the presence of underlying cardiovascular diseases and aging [35] in the control group, as shown in Table 1. Furthermore, the mentioned study [33] revealed similar findings to the current study with regard to cardiomyocyte diameter. Finally, a higher complexity of AF, as detected on wave maps, was associated with a higher amount of H&E endomysial fibrosis in LA using the AF goat model [36], suggesting a relationship between fibrosis and AF activation patterns. Additionally, another pre-clinical study revealed increased endomysial fibrosis in long-term (six months) AF goats in comparison to the short-term group [37]. However, comparable perimysial fibrosis between short-term, long-term, and the control group [37], which is in line with our findings.

In contrast with the tachypaced canine AF model due to underlying heart failure [38], we did not observe a higher expression of αSMA in human RAA in patients with history of AF compared to the control group. In line with that, the level of TIMP in RAA measured was comparable between all groups, similar to a previous clinical study that observed comparable TIMP levels in the LAA and RAA of AF and non-AF patients [39]. Although clinical and pre-clinical study revealed an overexpression of the early fibrosis marker NCAM in human hearts with ischemic cardiomyopathy and in experimental rat ischemic cardiomyopathy model [40], we observed comparable NCAM levels in RAA between patients with and without a history of AF. Whereas levels of LOX expression were higher in LAA from AF patients when compared to the controls [41], similar to a previous clinical study [42], we also observed comparable LOX levels in RAA and LAA among various stages of AF and the control group. Serum ratio CITP:MMP1 was presented as a strong biomarker relating myocardial fibrosis in patients with heart failure and hypertension [43], however, in our current study, the serum ratio CITP:MMP1 was similar between different stages of AF and the control group. These findings suggest that the status of atrial fibrosis in RAA and LAA as well as serum samples is comparable between different stages of AF and bring awareness to other factors responsible for the substrate underlying electropathology and AF.

### 4.2. Electrophysiology and Atrial Fibrosis in RAA

The unique mapping methodology utilized includes long duration multi-site high-resolution mapping of the LAA and RAA epicardial surface [21]. Our mapping approach was able to evaluate arrhythmogenic electrophysiological parameters both in the spatial and the temporal domain. This approach allows for localization and quantification of the degree of electropathology, providing a better understanding of the AF substrate in individual patients. Areas of atrial fibrosis are commonly considered to present slower local conduction velocity by influencing cardiomyocyte coupling and anisotropy [4,44]. Additionally, low voltage is often observed in ablated fibrotic sites [28,45]. However, so far, a direct correlation between high resolution electrophysiological parameters and the degree of fibrosis in the same region of human tissue has not been studied. Here, we correlated histological parameters (total, endomysial and epimysial fibrosis, and cardiomyocyte hypertrophy) with conduction abnormalities, conduction velocity, and voltage in RAA. No significant correlation was observed between histological and electrophysiological endpoints in absolute values, suggesting that atrial fibrosis in RAA is not directly and exclusively related to the degree of electropathology.

### 4.3. Spatial Analyses of Atrial Fibrosis: Histological and Electrophysiological Approach

Although clear electrical conduction abnormalities were observed in the control and AF samples, we did not observe an overlap in the percentage of total amount of fibrosis with low voltage area, nor with CB and/or CBCD lines. Here, data on spatial analyses compared two 2D planes in contiguity (i.e., histological and mapped epicardium) in human atrial tissue. By using this innovative approach, we provide final evidence that fibrosis is not underlying electrophysiological impairment in RAA and LAA. Further research is warranted to confirm the absence of a culprit role for fibrosis in electropathology in other atrial locations.

To date, areas with low-voltage have been recognized as surrogate markers for fibrosis and indicators of the arrhythmogenic substrate, thereby commonly guiding electrophysiologists on decision making for the identification of suitable target sites for ablation [4]. As the current study shows a lack of correlation between unipolar low-voltage areas and histological exuberant fibrotic status during sinus rhythm, the rationale behind these procedures is questionable.

Together, as our findings indicate that fibrosis is not the unique culprit factor driving electropathology and AF, we advocate for exploring new endpoints for structural remodeling.

### 4.4. Limitations

All patients in this study underwent cardiothoracic surgery for underlying heart disease. Together with aging [35], this fact may explain the already present degree of fibrosis in the control group. Although our patients were systematically classified based on available electrocardiograms (ECG), continuous cardiac rhythm monitoring would be more accurate to stage AF. As no changes in the degree of fibrosis was observed, re-classification of AF patients will not affect our conclusion. Due to ethical reasons, only LAA and/or RAA tissue samples were included in the current studies. Therefore, the findings cannot be extrapolated to other locations of the atria.

## Figures and Tables

**Table 1:** Patients characteristics.

	Control	ParAF	PerAF	LSPerAF	AF Total	*p* *	*p* †
Number of patients	48	22	28	17	67		
Male	34 (70.8)	15 (68.2)	19 (67.9)	15 (88.2)	49 (73.1)		
Age (years)	67.20 ± 12.12	68.28 ± 12.95	66.58 ± 8.54	71.80 ± 5.85	68.47		
BMI	27.92 (20.42–38.2)	25.23 (18.81–35.66)	26.47 (19.25–37.56)	28.37 (23.84–34.72)	26.45 (18.81–37.56)		
Hypertension	31 (64.6)	17 (77.3)	16 (57.1)	10 (58.8)	43 (64.2)		
Dyslipidemia	19 (39.6)	11 (50)	6 (21.4)	3 (17.6)	20 (29.9)		
DM	15 (31.3)	2 (9.1)	4 (14.3)	6 (35.3)	12 (17.9)		
Thyroid disease	4 (8.3)	3 (13.6)	2 (7.1)	1 (5.9)	6 (9)		
CHD	2 (4.2)	3 (13.6)	5 (17.9)	1 (5.9)	9 (13.4)		
AVD	4 (8.3)	4 (18.2)	5 (17.9)	3 (17.6)	12 (17.9)	0.0221	
CABG	29 (60.4)	4 (18.2)	2 (7.1)	4 (23.5)	10 (14.9)		
MVD	2 (4.2)	3 (13.6)	12 (42.9)	5 (9.4)	20 (29.9)		0.0156
MAZE	0	2 (9.1)	0	0	2 (3)		
AVD + CABG	9 (18.8)	4 (18.2)	2 (7.1)	2 (11.8)	8 (11.9)		
MVD + CABG-TVR	2 (4.2) 0	2 (9.1) 0	2 (7.1) 0	1 (5.9) 1 (5.9)	5 (7.5) 1 (1.5)		
LV Function							
-normal	39 (81.3)	19 (86.4)	16 (57.1)	10 (58.8)	45 (67.2)		
-mild imp.	9 (18.8)	2 (9.1)	6 (21.4)	5 (29.4)	13 (19.4)		
-moderate imp.	0	1 (4.5)	5 (17.9)	2 (11.8)	8 (11.9)		
-severe imp.	0	0	1 (3.6)	0	1 (1.5)		
Use of anti-arrhythmic drug							
-Class I	1 (2.1)	2 (9.1)	1 (3.6)	0	3 (4.5)		
-Class II	33 (68.8)	12 (54.5)	20 (71.4)	14 (82.4)	46 (68.7)		
-Class III	0	7 (31.8)	5 (17.9)	1 (5.9)	13 (19.4)	7 × 10^−4^	3.3×10^−3^
-Class IV	3 (6.3)	0	1 (3.6)	2 (11.8)	3 (4.5)		
Digoxin	0	1 (4.5)	9 (32.1)	5 (29.4)	15 (22.4)	7.8 × 10^−5^	1.2 × 10^−3^
Use of statin	35 (72.9)	13 (59.1)	9 (32.1)	12 (70.6)	34 (50.7)		
Use ACE, ARB, AT2 antagonist	30 (62.5)	12 (54.5)	18 (64.3)	14 (82.4)	44 (65.7)	4.2 × 10^−3^	2.8 × 10^−2^
-missing value				1			

Values are presented as N (%), mean ± SD or median (min-max). AF Total = any history of AF, ACE = angiotensin converting enzyme, ARB = angiotensin receptor blockers, AT2 = angiotensin II receptors, AVD = aortic valve disease, BMI = body mass index, CABG = coronary artery bypass grafting, CHD = congenital heart disease, DM = diabetes mellitus, imp. = impairment, MAZE = arrhythmia surgery, MVD = mitral valve disease, * = control/Type AF, † = control/Total AF. Statistical test: Pearson’s Chi-squared test and Yates’ continuity correction.

## Supplementary Materials

The following are available online at https://www.mdpi.com/article/10.3390/cells11030427/s1, Table S1: Clinical characteristics of patients included in the quantification of the total amount of fibrosis (picrosirius red), Table S2: Clinical characteristics of patients included in the quantification of cardiomyocyte hypertrophy, Table S3: Clinical characteristics of patients included in the quantification of endomysial and perimysial fibrosis, Table S4: Clinical characteristics of patients included in quantification of αSMA and TIMP expressions, Table S5: Clinical characteristics of patients included in the quantification of NCAM and LOX expressions, Table S6: Clinical characteristics of patients included in serum ratio CITP:MMP1 analysis, Table S7: Quantification of percentage of fibrosis in respect of Supplementary Figure S1, Table S8: Quantification of percentage of fibrosis with respect to Supplementary Figure S2, Table S9: Quantification of percentage of fibrosis with respect to Supplementary Figure S3, Table S10: Fibrosis parameters in right atrial appendages using western blot, Table S11: Serum CITP:MMP1 ratio, Table S12: Histological parameters for fibrosis, Table S13: Electrophysiological parameters, Figures S1–S5.
