# Supplementary material for: Degree of Fibrosis in Human Atrial Tissue Is Not the Hallmark Driving AF
Source: Cells. 2022 Jan 26;11(3):427. doi: 10.3390/cells11030427 (PMC8834228; doi:10.3390/cells11030427)
Supplement: Supplementary file 1 [file cells-11-00427-s001.zip › cells-1534238-supplementary.pdf]

Supplemental Material

Table S1. Clinical characteristics of patients included in quantification of total amount of fibrosis (picrosirius red).

Number of patients	62
Male	41 (66.1)
Age (years)	66.6774±12.07867
BMI	26.78 (19.11-38.2)
Type of AF	
- Paroxysmal	13 (21)
- Persistent	18 (29)
- Longstanding persistent	13 (21)
Hypertension	42 (67.7)
Dyslipidemia	22 (35.5)
Diabetes Mellitus	13 (21)
Thyroid disease	7 (11.3)
Underlying Cardiac Diseases	
- Congenital heart disease (CHD)	6 (9.7)
- Aortic valve disease (AVD)	9 (14.5)
- Coronary artery bypass grafting (CABG)	16 (25.8)
- Mitral valve disease (MVD)	14 (22.6)
- MAZE	4 (6.5)
- AVD + CABG	9 (14.5)
- MVD + CABG	4 (6.5)

Values are presented as N (%), mean ± SD or median (min-max). BMI = body mass index, LA = left atrium, RA = right atrium.

Table S2. Clinical characteristics of patients included in quantification of cardiomyocyte hypertrophy.

Number of patients	41
Male	25 (61)
Age (years)	65.3171±11.04183
BMI	26.81 (20.42-38.20)
Type of AF	
- Paroxysmal	9 (22)
- Persistent	12 (29.3)
- Longstanding persistent	8 (19.5)
Hypertension	27 (65.9)
Dyslipidemia	15 (36.6)
Diabetes Mellitus	8 (19.5)
Thyroid disease	4 (9.8)
Underlying Cardiac Diseases	
- Congenital heart disease (CHD)	4 (9.8)
- Aortic valve disease (AVD)	6 (14.6)
- Coronary artery bypass grafting (CABG)	8 (19.5)
- Mitral valve disease (MVD)	10 (24.4)
- MAZE	4 (9.8)
- AVD + CABG	6 (14.6)
- MVD + CABG	3 (7.3)

Values are presented as N (%), mean ± SD or median (min-max). BMI = body mass index, LA = left atrium, RA = right atrium.

Table S3. Clinical characteristics of patients included in quantification of endomysial and perimysial fibrosis.

Number of patients	29
Male	18 (62.1)
Age (years)	63.8966 ±11.65
BMI	25.95 (22.15-36.33)
Type of AF	
- Paroxysmal	8 (27.6)
- Persistent	8 (27.6)
- Longstanding persistent	5 (17.2)
Hypertension	18 (62.1)
Dyslipidemia	11 (37.9)
Diabetes Mellitus	3 (10.3)
Thyroid disease	3 (10.3)
Underlying Cardiac Diseases	
- Congenital heart disease (CHD)	4 (13.8)
- Aortic valve disease (AVD)	4 (13.8)
- Coronary artery bypass grafting (CABG)	3 (10.3)
- Mitral valve disease (MVD)	6 (20.7)
- MAZE	4 (13.8)
- AVD + CABG	5 (17.2)
- MVD + CABG	3 (10.3)

Values are presented as N (%), mean ± SD or median (min-max). BMI = body mass index, LA = left atrium, RA = right atrium.

Table S4. Clinical characteristics of patients included in quantification of αSMA and TIMP expressions.

Number of patients	90
Male	65 (72.2)
Age (years)	67.922 ±11.319
BMI	27.1100 (19.11-38.2)
Type of AF	
- Paroxysmal	13 (14.4)
- Persistent	20 (22.2)
- Longstanding persistent	13 (14.4)
Hypertension	62 (68.9)
Dyslipidemia	31 (34.4)
Diabetes Mellitus	22 (24.4)
Thyroid disease	9 (10)
Underlying Cardiac Diseases	
- Congenital heart disease (CHD)	6 (6.7)
- Aortic valve disease (AVD)	12 (13.3)
- Coronary artery bypass grafting (CABG)	35 (38.9)
- Mitral valve disease (MVD)	15 (16.7)
- MAZE	4 (4.4)
- AVD + CABG	14 (15.6)
- MVD + CABG	4 (4.4)

Values are presented as N (%), mean ± SD or median (min-max). BMI = body mass index, LA = left atrium, RA = right atrium.

Table S5. Clinical characteristics of patients included in quantification of NCAM and LOX expressions.

Number of patients	75
Male	57 (76)
Age (years)	67.84 ±11.47858
BMI	26.7500 (19.11-38.2)
Type of AF	
- Paroxysmal	9 (12)
- Persistent	17 (22.7)
- Longstanding persistent	11 (14.7)
Hypertension	50 (66.7)
Dyslipidemia	17 (22.7)
Diabetes Mellitus	17 (22.7)
Thyroid disease	8 (10.7)
Underlying Cardiac Diseases	
- Congenital heart disease (CHD)	6 (8)
- Aortic valve disease (AVD)	8 (10.7)
- Coronary artery bypass grafting (CABG)	31 (41.3)
- Mitral valve disease (MVD)	12 (16)
- MAZE	3 (4)
- AVD + CABG	12 (16)
- MVD + CABG	3 (4)

Values are presented as N (%), mean ± SD or median (min-max). BMI = body mass index, LA = left atrium, RA = right atrium.

Table S6. Clinical characteristics of patients included in serum ratio CITP:MMP1 analysis.

Number of patients	112
Male	81 (72.3)
Age (years)	67.9732 ±10.82245
BMI	26.735 (18.81-38.2)
Type of AF	
- Paroxysmal	20 (17.9)
- Persistent	27 (24.1)
- Longstanding persistent	18 (16.1)
Hypertension	73 (65.2)
Dyslipidemia	38 (33.9)
Diabetes Mellitus	26 (23.2)
Thyroid disease	9 (8)
Underlying Cardiac Diseases	
- Congenital heart disease (CHD)	11 (9.8)
- Aortic valve disease (AVD)	15 (13.4)
- Coronary artery bypass grafting (CABG)	38 (33.9)
- Mitral valve disease (MVD)	21 (18.8)
- MAZE	3 (2.7)
- AVD + CABG	17 (15.2)
- MVD + CABG	6 (5.4)

Values are presented as N (%), mean ± SD or median (min-max). BMI = body mass index, LA = left atrium, RA = right atrium.

Figure S1. Left atrial appendage I stained with Picrosirius Red.

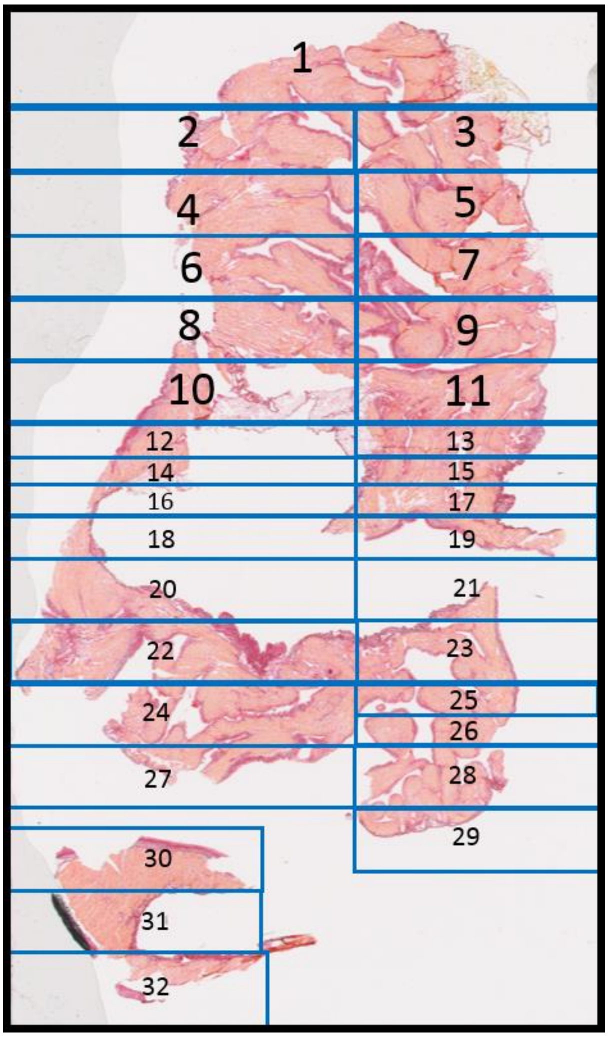

Table S7. Quantification of percentage of fibrosis in respect of supplementary figure S1

**Selection area**	**Amount of fibrosis (%)**
1	46,2438
2	45,2483
3	50,5229
4	42,9748
5	47,1053
6	37,0059
7	42,9544
8	47,3107
9	51,4942
10	48,6954
11	48,6917
12	54,419
13	51,7132
14	46,8222
15	52,1406
16	58,0279
17	42,4086
18	50,6694
19	57,078
20	37,764
21	48,6682
22	56,6255

23	56,7527
24	51,1785
25	53,4282
26	44,7798
27	49,0972
28	45,6073
29	44,5044
30	43,7838
31	41,3124
32	52,04

Figure S2. Left atrial appendage II stained with Picrosirius Red.

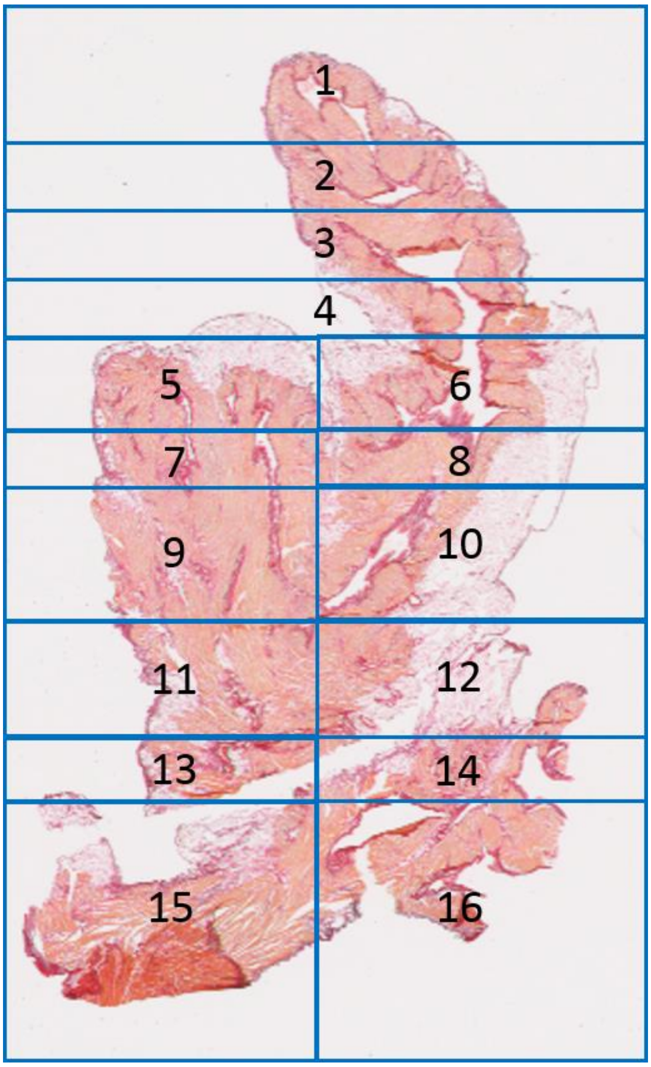

Table S8. Quantification of percentage of fibrosis in respect of supplementary figure S2.

Selection area	Amount of fibrosis (%)
1	68,8
2	64,0691
3	56,1891
4	61,5491
5	65,3478
6	64,3883
7	58,068
8	55,3248
9	54,7683
10	58,5337
11	53,2383
12	56,4717
13	68,233
14	70,0463
15	57,7396
16	60,8296

Figure S3. Left atrial appendage III stained with Picrosirius Red.

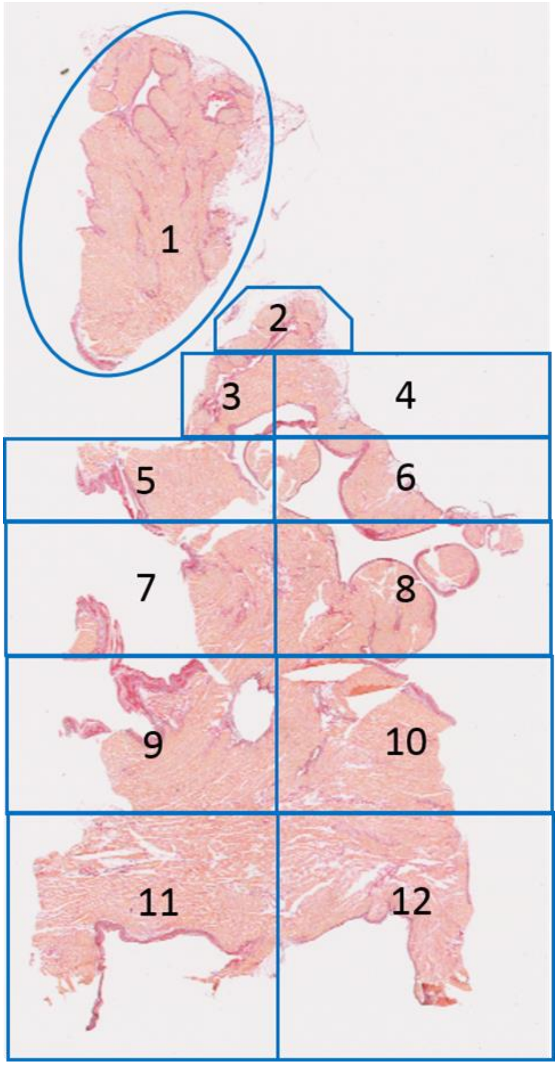

Table S9. Quantification of percentage of fibrosis in respect of supplementary figure S3.

**Selection area**	**Amount of fibrosis (%)**
1	45,8856
2	57,4743
3	49,8173
4	49,0287
5	46,4721
6	53,5367
7	45,908
8	45,5966
9	51,4198
10	44,6263
11	46,2378
12	49,4025

Figure S4. Blots of alpha smooth muscle actin (A) and its (B) GAPDH (B); (C) lysyl oxidase and its (D) GAPDH; (E) neural cell adhesion molecule and its (F) GAPDH; (G) tissue inhibitor of metalloproteinase and its (H) GAPDH. C = control, LAA = left atrial appendage, LSPerAF = Long standing persistent atrial fibrillation, ParAF = paroxysmal atrial fibrillation, PerAF = persistent atrial fibrillation, RAA = right atrial fibrillation, REF = reference band.

A

C	C	C	C	PerAF	PerAF	PerAF	C	PerAF	LSPerAF	PerAF	PerAF	C	C	C	REF
	RAA	RAA	RAA	RAA	LAA	RAA	RAA	RAA	RAA	RAA	LAA	RAA	RAA	RAA	

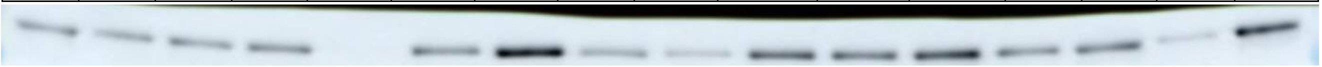

C	C	C	C	C	C	C		ParAF	ParAF	ParAF	ParAF	ParAF	LSPerAF	LSPerAF	REF
	RAA	RAA	RAA	RAA	RAA	RAA	RAA	RAA	RAA	LAA	RAA	RAA	RAA	RAA	RAA

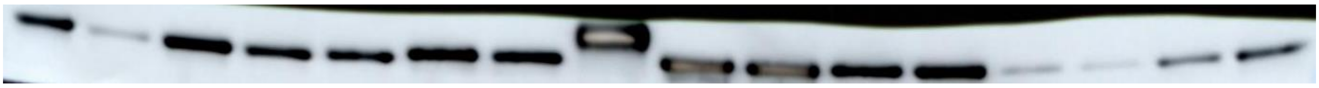

PerAF	PerAF	PerAF	ParAF	LSPerAF	LSPerAF	ParAF	ParAF	LSPerAF	LSPerAF		LSPerAF	LSPerAF	PerAF	C	REF
RAA	RAA	LAA	RAA	RAA	LAA	RAA	LAA	RAA	RAA		RAA	LAA	RAA	RAA	

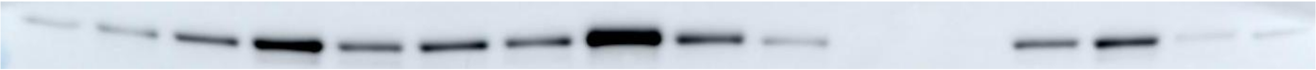

LSPerAF	LSPerAF	PerAF	PerAF	ParAF	ParAF	LSPerAF	ParAF	C	PerAF	PerAF	ParAF	PerAF	PerAF	C	REF
RAA	LAA	RAA	LAA	RAA	LAA		LAA	RAA	RAA	LAA	RAA	RAA	LAA	RAA	

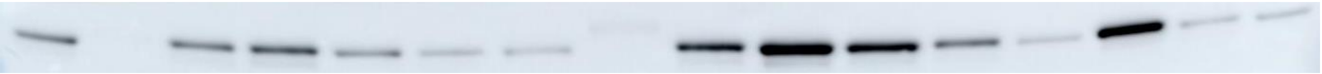

C	LSPerAF	C	ParAF	PerAF	LSPerAF	LSPerAF	LSPerAF	LSPerAF	C	PerAF	ParAF	PerAF	PerAF	C	REF
	LAA	RAA	RAA	RAA	LAA	RAA	LAA	RAA	RAA	LAA	RAA	RAA	LAA	LAA	

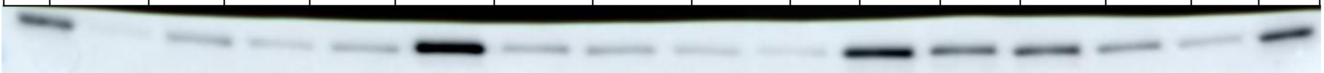

C	PerAF	C	C	PerAF	C	LSPerAF	C	C	LSPerAF	LSPerAF	C	PerAF	PerAF	ParAF	REF
RAA	RAA	RAA	RAA	LAA	RAA	LAA	RAA	RAA	RAA	LAA	RAA	RAA	LAA	RAA	

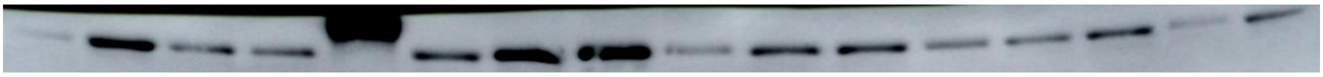

PerAF	PerAF	C	ParAF	ParAF	PerAF	LSPerAF	ParAF	PerAF	PerAF	C	PerAF	PerAF	PerAF	PerAF	REF
LAA	RAA	RAA	RAA	RAA	RAA	RAA	LAA	RAA	LAA	RAA	RAA	LAA	RAA	LAA	

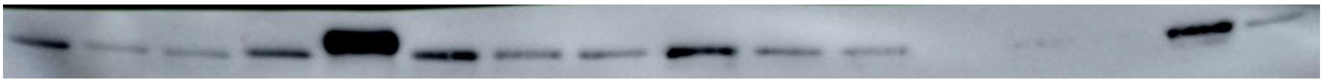

PerAF	C	C	C	PerAF	PerAF	PerAF	PerAF	PerAF	ParAF	ParAF	ParAF	C	C		REF
LAA	RAA	RAA	RAA	RAA	RAA	RAA	LAA	LAA	RAA	RAA	LAA	RAA	RAA		

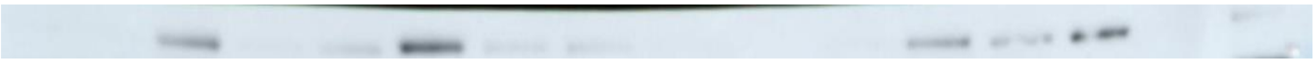

B

C	C	C	C	PerAF	PerAF	PerAF	C	PerAF	LSPerAF	PerAF	PerAF	C	C	C	REF
	RAA	RAA	RAA	RAA	LAA	RAA	RAA	RAA	RAA	RAA	LAA	RAA	RAA	RAA	

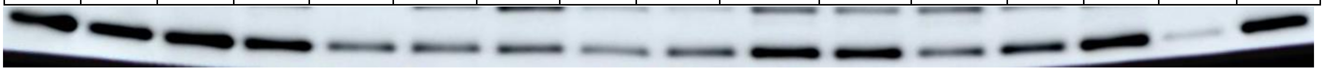

C	C	C	C	C	C	C		ParAF	ParAF	ParAF	ParAF	ParAF	LSPerAF	LSPerAF	REF
	RAA	RAA	RAA	RAA	RAA	RAA	RAA	RAA	RAA	LAA	RAA	RAA	RAA	RAA	RAA

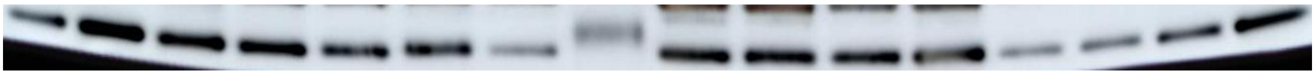

PerAF	PerAF	PerAF	ParAF	LSPerAF	LSPerAF	ParAF	ParAF	LSPerAF	LSPerAF		LSPerAF	LSPerAF	PerAF	C	REF
RAA	RAA	LAA	RAA	RAA	LAA	RAA	LAA	RAA	RAA		RAA	LAA	RAA	RAA	

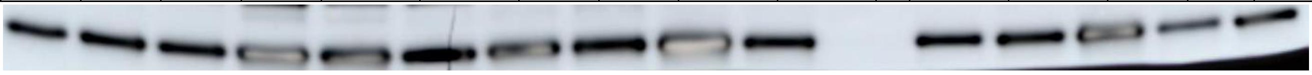

LSPerAF	LSPerAF	PerAF	PerAF	ParAF	ParAF	LSPerAF	ParAF	C	PerAF	PerAF	ParAF	PerAF	PerAF	C	REF
RAA	LAA	RAA	LAA	RAA	LAA		LAA	RAA	RAA	LAA	RAA	RAA	LAA	RAA	

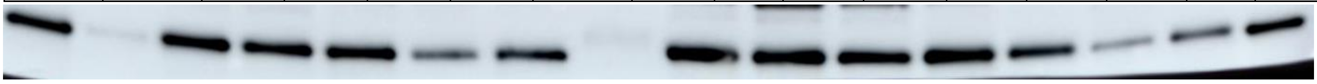

C	LSPerAF	C	ParAF	PerAF	LSPerAF	LSPerAF	LSPerAF	LSPerAF	C	PerAF	ParAF	PerAF	PerAF	C	REF
	LAA	RAA	RAA	RAA	LAA	RAA	LAA	RAA	RAA	LAA	RAA	RAA	LAA	LAA	

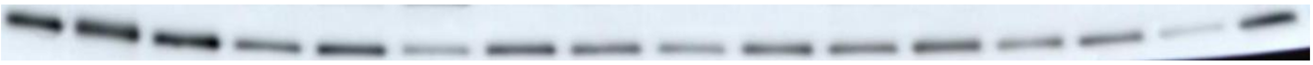

C	PerAF	C	C	PerAF	C	LSPerAF	C	C	LSPerAF	LSPerAF	C	PerAF	PerAF	ParAF	REF
RAA	RAA	RAA	RAA	LAA	RAA	LAA	RAA	RAA	RAA	LAA	RAA	RAA	LAA	RAA	

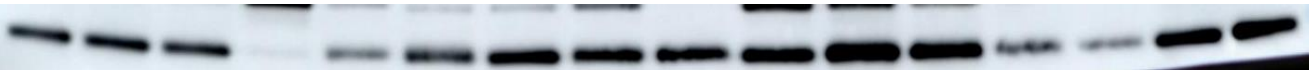

PerAF	PerAF	C	ParAF	ParAF	PerAF	LSPerAF	ParAF	PerAF	PerAF	C	PerAF	PerAF	PerAF	PerAF	REF
LAA	RAA	RAA	RAA	RAA	RAA	RAA	LAA	RAA	LAA	RAA	RAA	LAA	RAA	LAA	

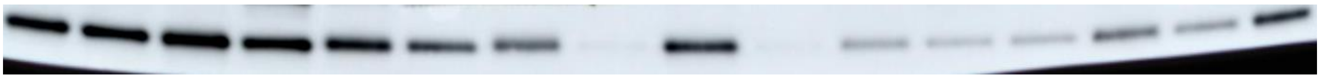

REF	C	C	LSPerAF	C	PerAF	PerAF	ParAF	C	PerAF	PerAF	C	C	C	C	C
	RAA	LAA	RAA	RAA	LAA	RAA	RAA	RAA	LAA	RAA	RAA	RAA	RAA	RAA	RAA

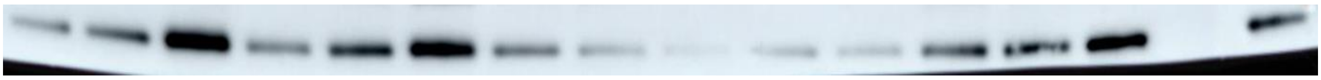

C

PerAF	C	C	C	C	LSPerAF	C	C	C	C	C	C	C	C	C	REF
LAA	RAA	RAA	RAA	RAA	RAA	RAA	RAA	RAA	RAA	RAA	RAA	RAA	RAA	RAA	

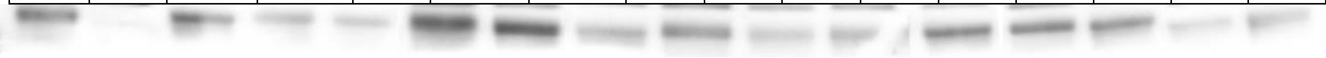

C	C	C	C	C	PerAF	PerAF	C	ParAF	PerAF	PerAF	C	LSPerAF	LSPerAF	C	REF
RAA	RAA	RAA	RAA	RAA	RAA	LAA	RAA	RAA	RAA	LAA	RAA	RAA	LAA	RAA	

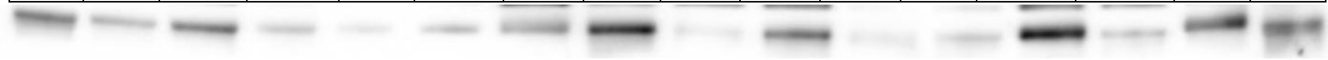

C	PerAF	PerAF	C	C	LSPerAF	PerAF	C	PerAF	LSPerAF	LSPerAF	LSPerAF	ParAF	ParAF	C	REF
	RAA	RAA	LAA	RAA	RAA	RAA	RAA	RAA	LAA	RAA	LAA	RAA	LAA	RAA	

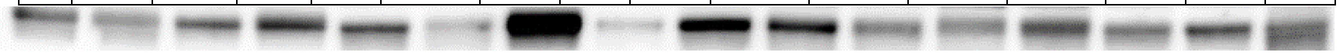

PerAF	C	C	PerAF	C	LSPerAF	C	C	LSPerAF	LSPerAF	C	PerAF	PerAF	PerAF	ParAF	REF
RAA	RAA	RAA	LAA	RAA	LAA	RAA	RAA	LAA	RAA	RAA	RAA	LAA	RAA	RAA	

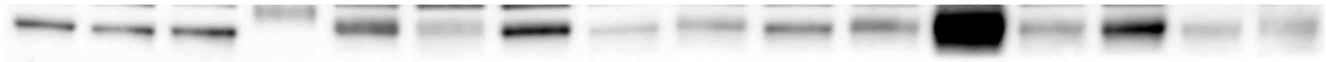

PerAF	PerAF	LSPerAF	LSPerAF	ParAF	ParAF	ParAF	LSPerAF	C	LSPerAF	C	LSPerAF	LSPerAF	LSPerAF	LSPerAF	REF
RAA	LAA	RAA	LAA	RAA	RAA	LAA	RAA	RAA	RAA	RAA	RAA	LAA	RAA	LAA	

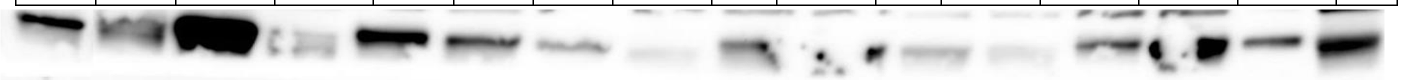

PerAF	C	PerAF	PerAF	ParAF	ParAF	ParAF	ParAF	C	PerAF	PerAF	ParAF	ParAF	PerAF	PerAF	REF
RAA	RAA	RAA	LAA	RAA	LAA	RAA	LAA	RAA	RAA	LAA	RAA	RAA	RAA	LAA	

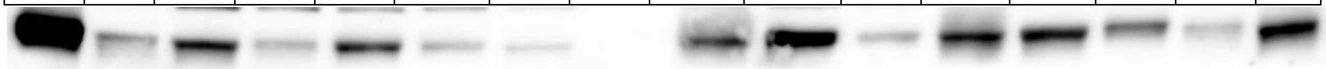

PerAF	PerAF	C	C	ParAF	ParAF	PerAF	LSPerAF	ParAF	PerAF	PerAF	C	PerAF	PerAF	C	REF
RAA	LAA	RAA	RAA	RAA	LAA	RAA	RAA	LAA	RAA	LAA	RAA	RAA	LAA	RAA	

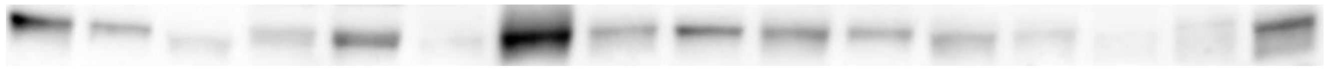

PerAF	PerAF	ParAF	C	ParAF	LSPerAF	LSPerAF	LSPerAF	ParAF	PerAF	PerAF	ParAF	ParAF	C	C	REF
RAA	LAA	RAA	RAA	RAA	RAA	LAA	RAA	RAA	RAA	LAA	RAA	LAA	RAA	RAA	

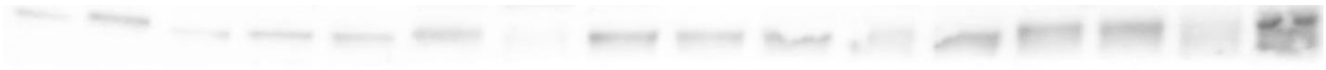

D

PerAF	C	C	C	C	LSPerAF	C	C	C	C	C	C	C	C	C	REF
LAA	RAA	RAA	RAA	RAA	RAA	RAA	RAA	RAA	RAA	RAA	RAA	RAA	RAA	RAA	

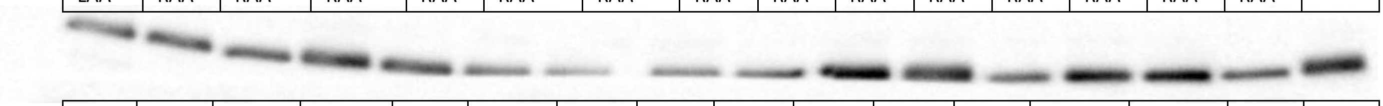

C	C	C	C	C	PerAF	PerAF	C	ParAF	PerAF	PerAF	C	LSPerAF	LSPerAF	C	REF
RAA	RAA	RAA	RAA	RAA	RAA	LAA	RAA	RAA	RAA	LAA	RAA	RAA	LAA	RAA	

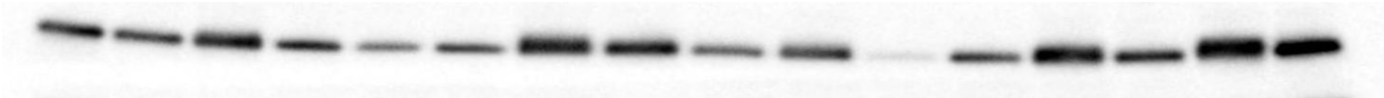

C	PerAF	PerAF	C	C	LSPerAF	PerAF	C	PerAF	LSPerAF	LSPerAF	LSPerAF	ParAF	ParAF	C	REF
	RAA	RAA	LAA	RAA	RAA	RAA	RAA	RAA	LAA	RAA	LAA	RAA	LAA	RAA	

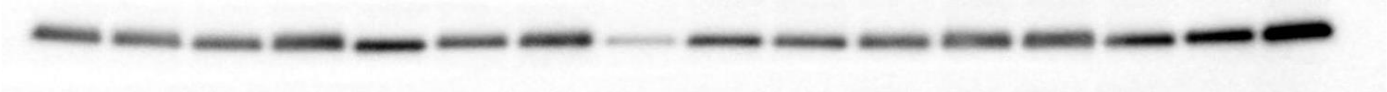

PerAF	C	C	PerAF	C	LSPerAF	C	C	LSPerAF	LSPerAF	C	PerAF	PerAF	PerAF	ParAF	REF
RAA	RAA	RAA	LAA	RAA	LAA	RAA	RAA	LAA	RAA	RAA	RAA	LAA	RAA	RAA	

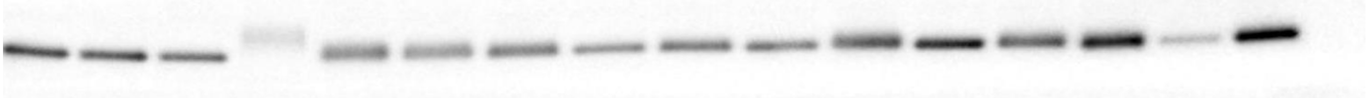

PerAF	PerAF	LSPerAF	LSPerAF	ParAF	ParAF	ParAF	LSPerAF	C	LSPerAF	C	LSPerAF	LSPerAF	LSPerAF	LSPerAF	REF
RAA	LAA	RAA	LAA	RAA	RAA	LAA	RAA	RAA	RAA	RAA	RAA	LAA	RAA	LAA	

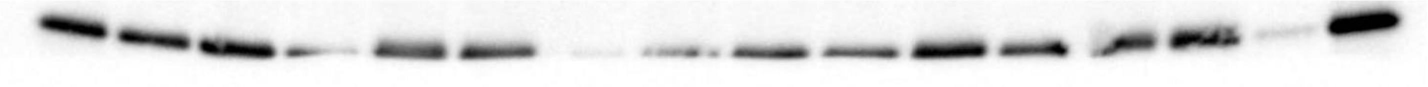

PerAF	C	PerAF	PerAF	ParAF	ParAF	PerAF	ParAF	C	PerAF	PerAF	ParAF	ParAF	PerAF	PerAF	REF
RAA	RAA	RAA	LAA	RAA	LAA	RAA	LAA	RAA	RAA	LAA	RAA	RAA	RAA	LAA	

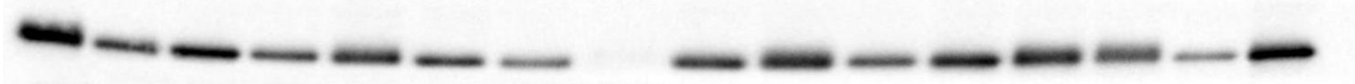

PerAF	PerAF	C	C	ParAF	ParAF	PerAF	LSPerAF	ParAF	PerAF	PerAF	C	PerAF	PerAF	C	REF
RAA	LAA	RAA	RAA	RAA	LAA	RAA	RAA	LAA	RAA	LAA	RAA	RAA	LAA	RAA	

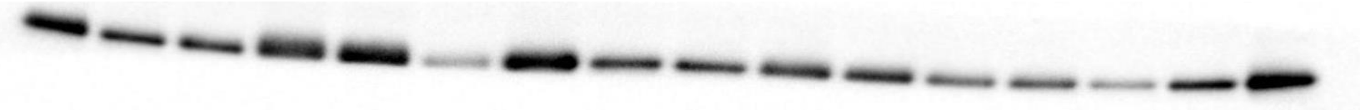

PerAF	PerAF	ParAF	C	ParAF	LSPerAF	LSPerAF	LSPerAF	ParAF	PerAF	PerAF	ParAF	ParAF	C	C	REF
RAA	LAA	RAA	RAA	RAA	RAA	LAA	RAA	RAA	RAA	LAA	RAA	LAA	RAA	RAA	

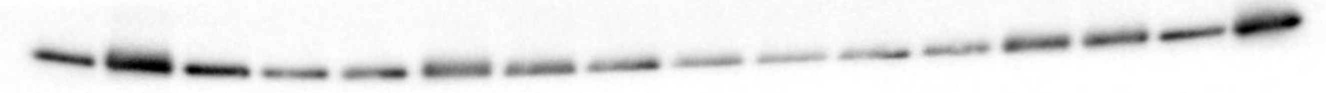

E

PerAF	C	C	C	C	LSPerAF	C	C	C	C	C	C	C	C	C	REF
LAA	RAA	RAA	RAA	RAA	RAA	RAA	RAA	RAA	RAA	RAA	RAA	RAA	RAA	RAA	

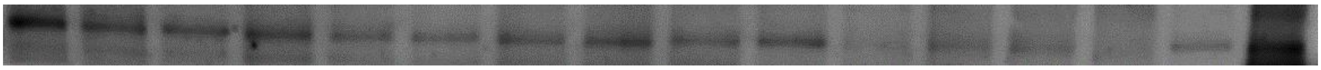

C	C	C	C	C	PerAF	PerAF	C	ParAF	PerAF	PerAF	C	LSPerAF	LSPerAF	C	REF
RAA	RAA	RAA	RAA	RAA	RAA	LAA	RAA	RAA	RAA	LAA	RAA	RAA	LAA	RAA	

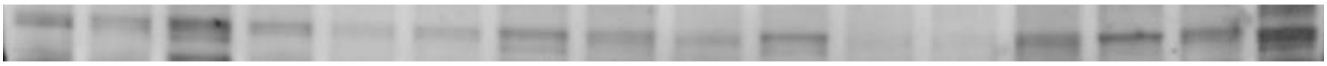

C	PerAF	PerAF	C	C	LSPerAF	PerAF	C	PerAF	LSPerAF	LSPerAF	LSPerAF	ParAF	ParAF	C	REF
	RAA	RAA	LAA	RAA	RAA	RAA	RAA	RAA	LAA	RAA	LAA	RAA	LAA	RAA	

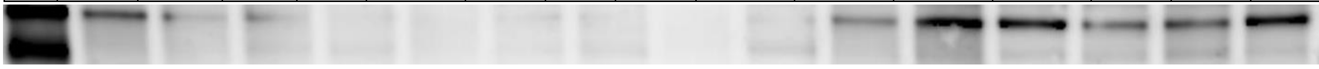

PerAF	C	C	PerAF	C	LSPerAF	C	C	LSPerAF	LSPerAF	C	PerAF	PerAF	ParAF	PerAF	REF
RAA	RAA	RAA	LAA	RAA	LAA	RAA	RAA	RAA	LAA	RAA	RAA	LAA	RAA	RAA	

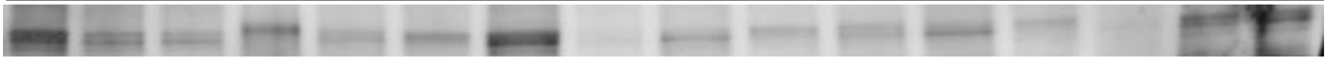

PerAF	PerAF	LSPerAF	LSPerAF	ParAF	ParAF	ParAF	LSPerAF	C	LSPerAF	C	LSPerAF	LSPerAF	LSPerAF	LSPerAF	REF
RAA	LAA	RAA	LAA	RAA	RAA	LAA	RAA	RAA	RAA	RAA	RAA	LAA	RAA	LAA	

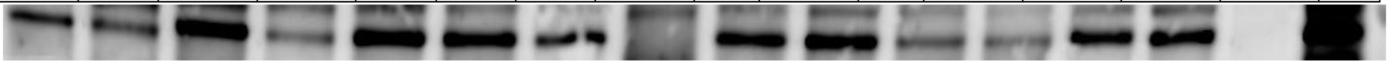

PerAF	C	PerAF	PerAF	ParAF	ParAF	ParAF	ParAF	C	PerAF	PerAF	ParAF	ParAF	PerAF	PerAF	REF
RAA	RAA	RAA	LAA	RAA	LAA	RAA	LAA	RAA	RAA	LAA	RAA	RAA	RAA	LAA	

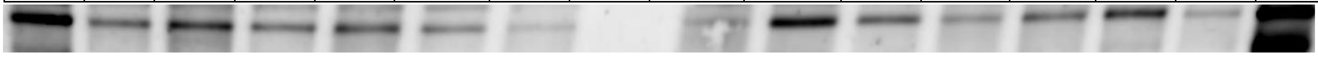

PerAF	PerAF	C	C	ParAF	ParAF	PerAF	LSPerAF	ParAF	PerAF	PerAF	C	PerAF	PerAF	C	REF
RAA	LAA	RAA	RAA	RAA	LAA	RAA	RAA	LAA	RAA	LAA	RAA	RAA	LAA	RAA	

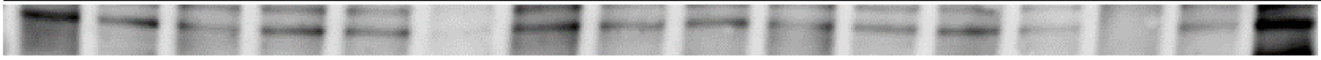

PerAF	PerAF	C	ParAF	LSPerAF	LSPerAF	ParAF	PerAF	PerAF	ParAF	ParAF	C	C	C	C	REF
RAA	LAA	RAA	RAA	RAA	RAA	RAA	RAA	LAA	RAA	LAA	RAA	RAA	RAA	LAA	

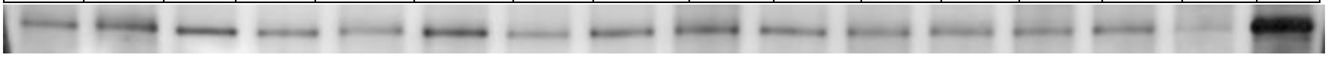

F

PerAF	C	C	C	C	LSPerAF	C	C	C	C	C	C	C	C	C	REF
LAA	RAA	RAA	RAA	RAA	RAA	RAA	RAA	RAA	RAA	RAA	RAA	RAA	RAA	RAA	

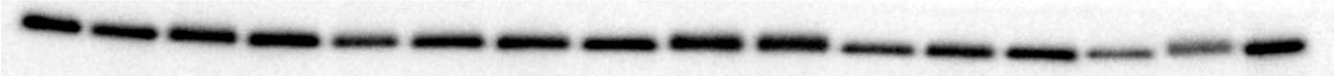

C	C	C	C	C	PerAF	PerAF	C	ParAF	PerAF	PerAF	C	LSPerAF	LSPerAF	C	REF
RAA	RAA	RAA	RAA	RAA	RAA	LAA	RAA	RAA	RAA	LAA	RAA	RAA	LAA	RAA	

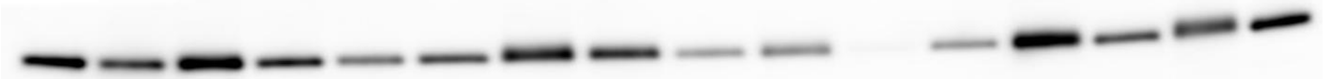

C	PerAF	PerAF	C	C	LSPerAF	PerAF	C	PerAF	LSPerAF	LSPerAF	LSPerAF	ParAF	ParAF	C	REF
	RAA	RAA	LAA	RAA	RAA	RAA	RAA	RAA	LAA	RAA	LAA	RAA	LAA	RAA	

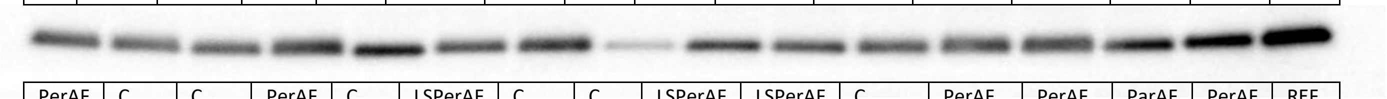

PerAF	C	C	PerAF	C	LSPerAF	C	C	LSPerAF	LSPerAF	C	PerAF	PerAF	ParAF	PerAF	REF
RAA	RAA	RAA	LAA	RAA	LAA	RAA	RAA	RAA	LAA	RAA	RAA	LAA	RAA	RAA	

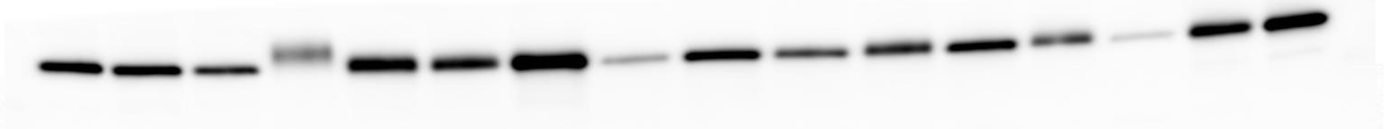

PerAF	PerAF	LSPerAF	LSPerAF	ParAF	ParAF	ParAF	LSPerAF	C	LSPerAF	C	LSPerAF	LSPerAF	LSPerAF	LSPerAF	REF
RAA	LAA	RAA	LAA	RAA	RAA	LAA	RAA	RAA	RAA	RAA	RAA	LAA	RAA	LAA	

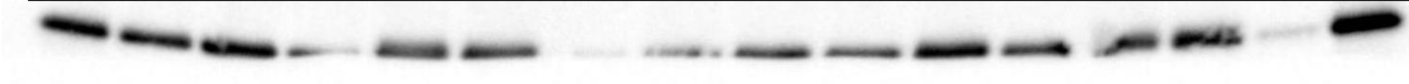

PerAF	C	PerAF	PerAF	ParAF	ParAF	ParAF	ParAF	C	PerAF	PerAF	ParAF	ParAF	PerAF	PerAF	REF
RAA	RAA	RAA	LAA	RAA	LAA	RAA	LAA	RAA	RAA	LAA	RAA	RAA	RAA	LAA	

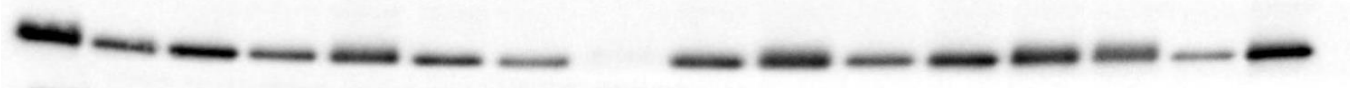

PerAF	PerAF	C	C	ParAF	ParAF	PerAF	LSPerAF	ParAF	PerAF	PerAF	C	PerAF	PerAF	C	REF
RAA	LAA	RAA	RAA	RAA	LAA	RAA	RAA	LAA	RAA	LAA	RAA	RAA	LAA	RAA	

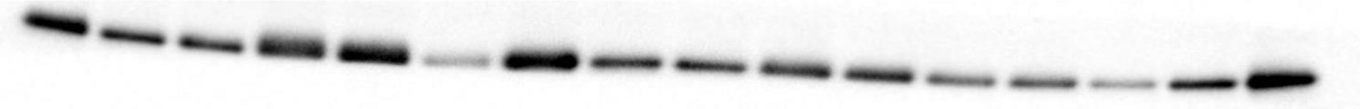

PerAF	PerAF	C	ParAF	LSPerAF	LSPerAF	ParAF	PerAF	PerAF	ParAF	ParAF	C	C	C	C	REF
RAA	LAA	RAA	RAA	RAA	RAA	RAA	RAA	LAA	RAA	LAA	RAA	RAA	RAA	LAA	

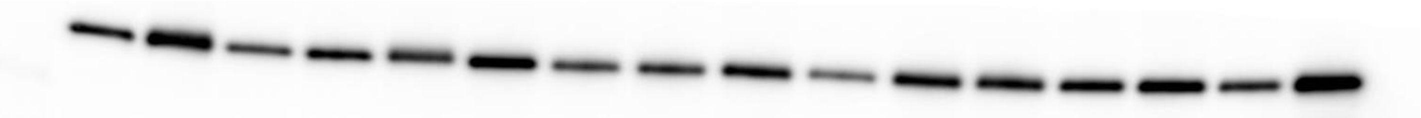

G

C	C	C	C	C	C	C	C	C	LSPerAF	C	C	C	C	PerAF	REF
RAA	RAA	RAA	RAA	RAA	RAA	RAA	RAA	RAA	RAA	RAA	RAA	RAA	RAA	LAA	

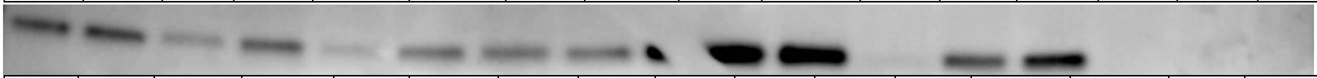

C	C	C	C	C	PerAF	PerAF	C	ParAF	PerAF	PerAF	C	LSPerAF	LSPerAF	C	REF
RAA	RAA	RAA	RAA	RAA	RAA	LAA	RAA	RAA	RAA	LAA	RAA	RAA	LAA	RAA	

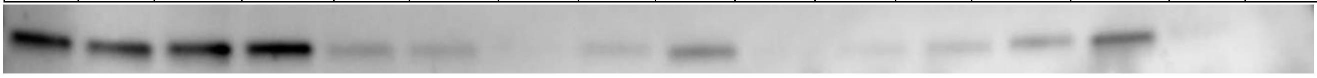

C	PerAF	PerAF	C	C	LSPerAF	PerAF	C	PerAF	LSPerAF	LSPerAF	LSPerAF	ParAF	ParAF	C	REF
	RAA	RAA	LAA	RAA	RAA	RAA	RAA	RAA	LAA	RAA	LAA	RAA	LAA	RAA	

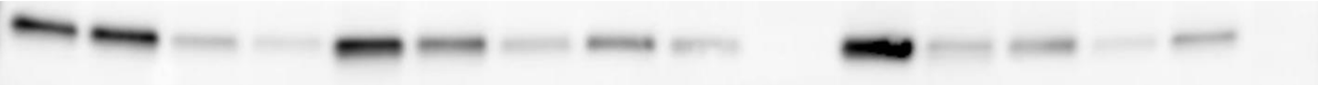

PerAF	C	C	PerAF	C	LSPerAF	C	C	LSPerAF	LSPerAF	C	PerAF	PerAF	ParAF	PerAF	REF
RAA	RAA	RAA	LAA	RAA	LAA	RAA	RAA	LAA	RAA	RAA	RAA	LAA	RAA	RAA	

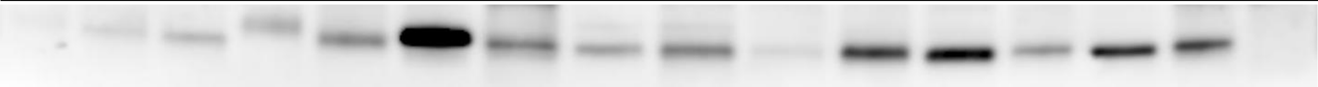

PerAF	PerAF	LSPerAF	LSPerAF	ParAF	ParAF	ParAF	LSPerAF	C	LSPerAF	C	LSPerAF	LSPerAF	PerAF	LSPerAF	REF
RAA	LAA	RAA	LAA	RAA	RAA	LAA	RAA	RAA	RAA	RAA	RAA	LAA	RAA	LAA	

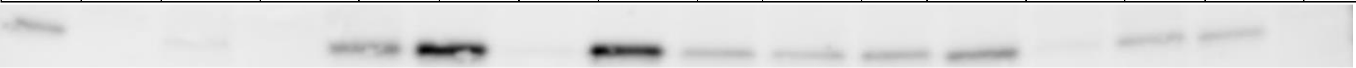

LSPerAF	LSPerAF	PerAF	PerAF	ParAF	ParAF	ParAF	ParAF	C	PerAF	PerAF	ParAF	ParAF	PerAF	PerAF	REF
RAA	LAA	RAA	LAA	RAA	LAA	RAA	LAA	RAA	RAA	LAA	RAA	RAA	RAA	LAA	

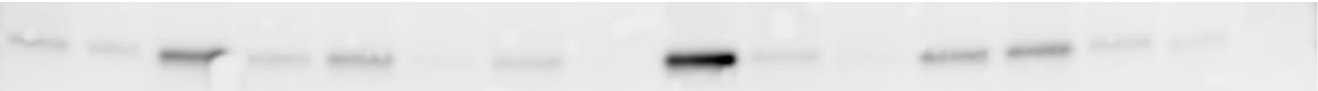

PerAF	PerAF	C	C	ParAF	ParAF	PerAF	LSPerAF	ParAF	PerAF	PerAF	C	PerAF	PerAF	ParAF	REF
RAA	LAA	RAA	RAA	RAA	LAA	RAA	RAA	LAA	RAA	LAA	RAA	RAA	LAA	RAA	

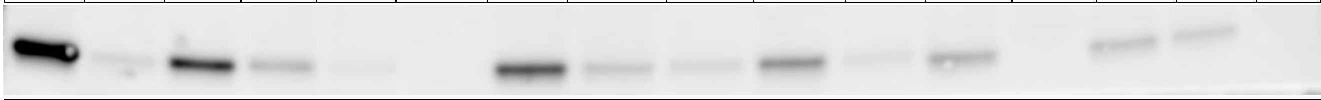

PerAF	PerAF	ParAF	C	ParAF	LSPerAF	LSPerAF	LSPerAF	ParAF	PerAF	PerAF	ParAF	ParAF	C	C	REF
RAA	LAA	RAA	RAA	RAA	RAA	LAA	RAA	RAA	RAA	LAA	RAA	LAA	RAA	RAA	

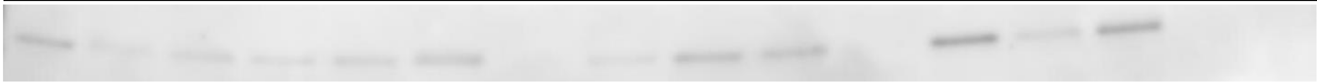

H

C	C	C	C	C	C	C	C	C	LSPerAF	C	C	C	C	PerAF	REF
RAA	RAA	RAA	RAA	RAA	RAA	RAA	RAA	RAA	RAA	RAA	RAA	RAA	RAA	LAA	

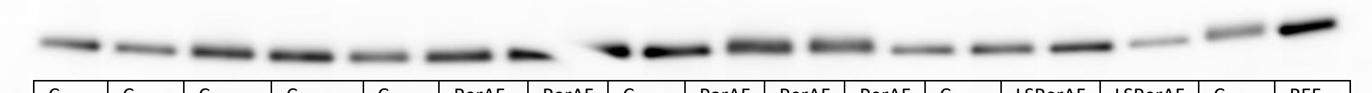

C	C	C	C	C	PerAF	PerAF	C	ParAF	PerAF	PerAF	C	LSPerAF	LSPerAF	C	REF
RAA	RAA	RAA	RAA	RAA	RAA	LAA	RAA	RAA	RAA	LAA	RAA	RAA	LAA	RAA	

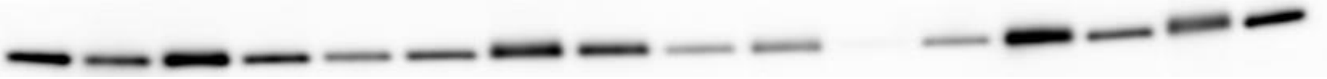

C	PerAF	PerAF	C	C	LSPerAF	PerAF	C	PerAF	LSPerAF	LSPerAF	LSPerAF	ParAF	ParAF	C	REF
	RAA	RAA	LAA	RAA	RAA	RAA	RAA	RAA	LAA	RAA	LAA	RAA	LAA	RAA	

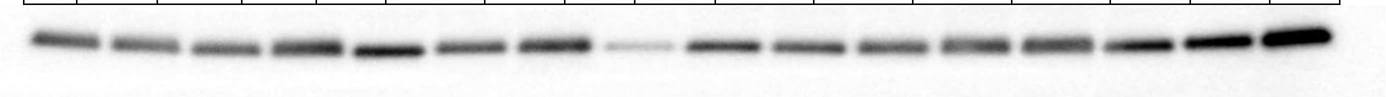

PerAF	C	C	PerAF	C	LSPerAF	C	C	LSPerAF	LSPerAF	C	PerAF	PerAF	ParAF	PerAF	REF
RAA	RAA	RAA	LAA	RAA	LAA	RAA	RAA	LAA	RAA	RAA	RAA	LAA	RAA	RAA	

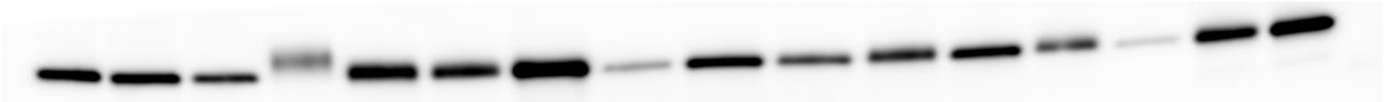

PerAF	PerAF	LSPerAF	LSPerAF	ParAF	ParAF	ParAF	LSPerAF	C	LSPerAF	C	LSPerAF	LSPerAF	PerAF	LSPerAF	REF
RAA	LAA	RAA	LAA	RAA	RAA	LAA	RAA	RAA	RAA	RAA	RAA	LAA	RAA	LAA	

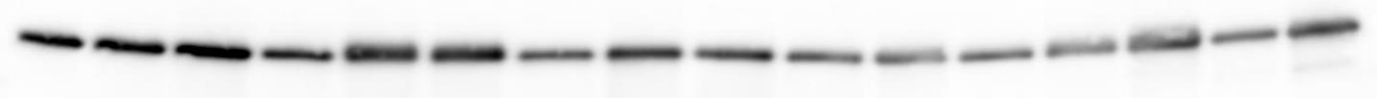

LSPerAF	LSPerAF	PerAF	PerAF	ParAF	ParAF	ParAF	ParAF	C	PerAF	PerAF	ParAF	ParAF	PerAF	PerAF	REF
RAA	LAA	RAA	LAA	RAA	LAA	RAA	LAA	RAA	RAA	LAA	RAA	RAA	RAA	LAA	

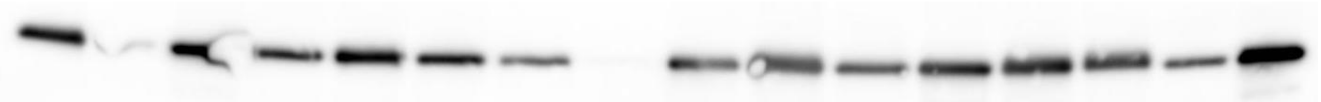

PerAF	PerAF	C	C	ParAF	ParAF	PerAF	LSPerAF	ParAF	PerAF	PerAF	C	PerAF	PerAF	ParAF	REF
RAA	LAA	RAA	RAA	RAA	LAA	RAA	RAA	LAA	RAA	LAA	RAA	RAA	LAA	RAA	

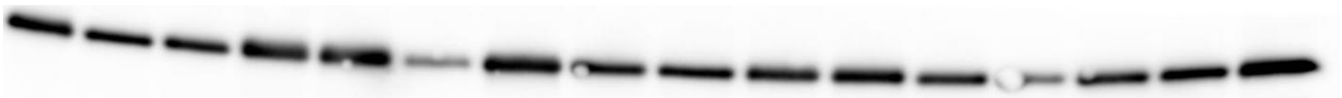

PerAF	PerAF	ParAF	C	ParAF	LSPerAF	LSPerAF	LSPerAF	ParAF	PerAF	PerAF	ParAF	ParAF	C	C	REF
RAA	LAA	RAA	RAA	RAA	RAA	LAA	RAA	RAA	RAA	LAA	RAA	LAA	RAA	RAA	

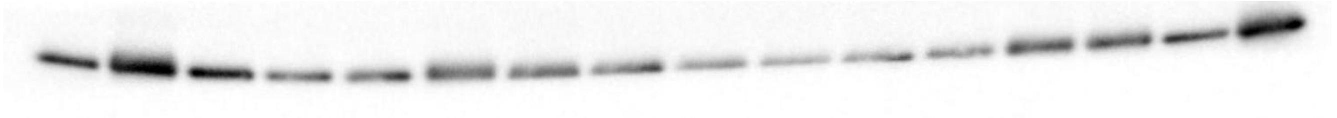

Figure S5. Degree of fibrosis and post-operative AF. Picrosirius red staining reveals comparable degree of fibrosis between patients with and without post-operative AF. PoAF = post-operative AF. P>0,05.

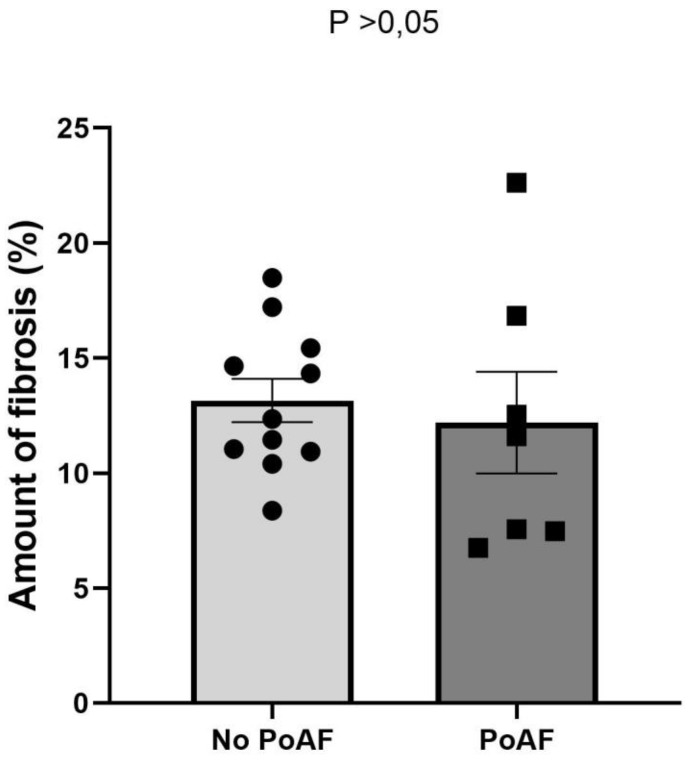

Table S10. Fibrosis parameters in right atrial appendages, using Western blot.

	α-SMA/GAPDH (arbitrary unit: a.u)	TIMP/GAPDH (a.u)	NCAM/GAPDH (a.u.)	LOX/GAPDH (a.u.)
Patient 1	0,135	0,993	0,74	0,65
Patient 2	0,129	1,247	0,693	1,42
Patient 3	1,114	0,504	0,558	0,8
Patient 4	0,113	0,551	0,675	1,27
Patient 5	0,295	0,396	1,172	1,04
Patient 6	0,332	0,659	0,709	0,85
Patient 7	0,413	0,463	0,618	0,65
Patient 8	0,065	0,676	0,446	1,26
Patient 9	0,787	1,113	0,617	1,11
Patient 10	0,084	1,905	0,472	1,25
Patient 11	0,165	0,574	0,627	0,83
Patient 12	0,639	1,855	0,662	0,71
Patient 13	0,906			
Patient 14	0,918	2,172	0,748	0,98
Patient 15	0,25	0,349	1,693	0,53
Patient 16	0,201	1,279	0,34	1,41
Patient 17	0,362	1,741	0,384	0,71
Patient 18	1,175	1,366	0,432	1,05
Patient 19	0,796	1,192	0,509	0,69
Patient 20	0,297	0,491	0,363	0,87
Patient 21	0,872	0,531	0,294	0,96

Patient 22	0,34	0,459	0,3	0,89
Patient 23	0,36	1,442	0,387	0,46
Patient 24	0,466	0,235	0,4	1,34
Patient 25	0,282	1,272	0,397	0,76
Patient 26	0,769	1,318	0,26	1,53
Patient 27	1,226	0,238	0,605	0,76
Patient 28	0,765	1,072	0,745	0,79
Patient 29	0,049	1,547	0,732	0,66
Patient 30	1,424	0,119	0,682	0,71
Patient 31	0,612	1,781	0,92	0,77
Patient 32	0,63	1,063	0,668	0,71
Patient 33	0,229	0,262	0,8	1,68
Patient 34	0,42	3,513		
Patient 35	0,603	0,269	0,755	1,19
Patient 36				1,2
Patient 37	1,918	3,466	0,899	
Patient 38	0,743	0,476	1,256	
Patient 39	0,223	0,234	0,852	1,08
Patient 40	1,273	0,082	0,782	
Patient 41	0,525	0,264	0,745	0,84
Patient 42	1,126	0,758	1,352	
Patient 43				0,61
Patient 44	0,754	0,839	0,614	0,48
Patient 45				0,49
Patient 46	0,981	0,433	0,884	1,03
Patient 47	0,459	2,029	0,781	

Patient 48	0,348	1,049	0,447	0,61
Patient 49	0,454	1,672	0,79	
Patient 50	0,477	1,531	0,765	0,54
Patient 51	0,418	4,65	0,544	0,43
Patient 52	0,408	0,956	1,052	0,93
Patient 53	0,326	0,752	0,43	
Patient 54	0,754	0,179	0,832	0,49
Patient 55	1,273	1,203	0,646	
Patient 56	0,957	2,494	0,59	2,89
Patient 57	0,809	2,225	0,38	1,29
Patient 58	1	0,487	0,52	0,87
Patient 59	0,496	0,332	0,778	2,03
Patient 60	0,23	0,428	0,304	
Patient 61	0,044	1,775	0,444	4,05
Patient 62	1,023	0,346	0,784	1,14
Patient 63	0,542	0,4	0,922	0,66
Patient 64	0,807	0,658	0,792	0,11
Patient 65	0,437	1,422	0,78	
Patient 66	1,287	0,724	0,799	0,49
Patient 67	0,244	0,818	0,619	0,44
Patient 68	0,649	2,181	1,1	0,31
Patient 69	1,733	0,392	1,137	
Patient 70	0,43	0,905	0,832	0,4
Patient 71	0,744	0,905	0,75	1,84
Patient 72	0,681	0,471	1,134	1,06
Patient 73	0,733	2,805	0,502	0,63

Patient 74	0,419	2,136	0,451	0,81
Patient 75	0,338	0,434	0,428	0,54
Patient 76	0,602	0,123	0,355	
Patient 77	0,471	1,1	0,423	0,27
Patient 78	0,668	0,435	0,534	1,48
Patient 79	1,132	0,756	0,449	1,04
Patient 80	1,078	0,373	0,627	1,23
Patient 81	0,208	0,496		0,76
Patient 82	0,521	0,609		0,54
Patient 83	0,098	0,313		
Patient 84	0,558	0,763		0,76
Patient 85	0,51	0,803		0,68
Patient 86	0,219	0,856		0,65
Patient 87	0,492	0,311		0,83
Patient 88	0,411	1,751		1,9
Patient 89	0,323	0,503		1
Patient 90	0,401	3,048		
Patient 91	1,102	1,871		1,01
Patient 92				0,72
Patient 93	1,018	0,203		0,53
Patient 94		0,248		
Patient 95	1,3			

Table S11. Serum CITP:MMP1 ratio.

Serum CITP:MMP1 ratio (a.u.)			
Patient 1	0,028	Patient 56	0,058
Patient 2	0,024	Patient 57	0,048
Patient 3	0,018	Patient 58	0,142
Patient 4	0,029	Patient 59	0,029
Patient 5	0,03	Patient 60	0,048
Patient 6	0,027	Patient 61	0,044
Patient 7	0,027	Patient 62	0,016
Patient 8	0,034	Patient 63	0,027
Patient 9	0,011	Patient 64	0,027
Patient 10	0,049	Patient 65	0,042
Patient 11	0,016	Patient 66	0,039
Patient 12	0,056	Patient 67	0,1
Patient 13	0,024	Patient 68	0,069
Patient 14	0,029	Patient 69	0,048
Patient 15	0,024	Patient 70	0,042
Patient 16	0,015	Patient 71	0,039
Patient 17	0,056	Patient 72	0,151
Patient 18	0,013	Patient 73	0,024
Patient 19	0,028	Patient 74	0,019
Patient 20	0,019	Patient 75	0,128
Patient 21	0,008	Patient 76	0,023
Patient 22	0,057	Patient 77	0,04

Patient 23	0,039	Patient 78	0,141
Patient 24	0,009	Patient 79	0,069
Patient 25	0,008	Patient 80	0,038
Patient 26	0,031	Patient 81	0,029
Patient 27	0,042	Patient 82	0,187
Patient 28	0,018	Patient 83	0,034
Patient 29	0,072	Patient 84	0,071
Patient 30	0,023	Patient 85	0,065
Patient 31	0,024	Patient 86	0,046
Patient 32	0,136	Patient 87	0,066
Patient 33	0,025	Patient 88	0,037
Patient 34	0,005	Patient 89	0,111
Patient 35	0,051	Patient 90	0,054
Patient 36	0,042	Patient 91	0,022
Patient 37	0,022	Patient 92	0,048
Patient 38	0,034	Patient 93	0,046
Patient 39	0,031	Patient 94	0,038
Patient 40	0,032	Patient 95	0,174
Patient 41	0,062	Patient 96	0,08
Patient 42	0,044	Patient 97	0,03
Patient 43	0,026	Patient 98	0,233
Patient 44	0,035	Patient 99	0,014
Patient 45	0,069	Patient 100	0,036
Patient 46	0,049	Patient 101	0,05
Patient 47	0,02	Patient 102	0,041
Patient 48	0,028	Patient 103	0,037

Patient 49	0,021	Patient 104	0,061
Patient 50	0,068	Patient 105	0,026
Patient 51	0,107	Patient 106	0,036
Patient 52	0,014	Patient 107	0,044
Patient 53	0,053	Patient 108	0,064
Patient 54	0,026	Patient 109	0,048
Patient 55	0,04	Patient 110	0,109

Table S12. Histological parameters for fibrosis.

	**Cardiomyocyte D. (μm)**	**Endomysial F. (μm)**	**Perimysium F. (μm)**	**Total fibrosis – Picrosirius (%)**
Patient 1	18,256			11,05
Patient 2				11,599
Patient 3				17,016
Patient 4				12,538
Patient 5	26,929			14,658
Patient 6	41,162	5,206	18,789	7,193
Patient 7	37,016	4,994	16,004	6,746
Patient 8	37,178	6,831	21,974	15,428
Patient 9	39,185	3,912	13,308	7,554
Patient 10	46,148	4,352	13,846	8,364
Patient 11	20,002			11,652
Patient 12	44,027	3,639	18,221	12,227
Patient 13	28,774	3,372	12,354	15,707

Patient 14				17,447
Patient 15	46,143			13,971
Patient 16				8,477
Patient 17				10,95
Patient 18	36,625	3,516	9,45	14,354
Patient 19	33,853	2,484	10,665	16,41
Patient 20				14,339
Patient 21				22,63
Patient 22	45,585	6,432	15,408	7,473
Patient 23	38,661	3,597	17,2	7,332
Patient 24				16,841
Patient 25	39,359	5,21	15,073	11,348
Patient 26	32,537	4,899	20,7	8,861
Patient 27	43,019	5,062	15,252	15,216
Patient 28	37,557			9,479
Patient 29	52,057	4,304	19,639	12,541
Patient 30	37,805	4,041	16,075	11,689
Patient 31	33,689	5,675	20,896	9,304
Patient 32				25,14
Patient 33	48,94			14,183
Patient 34				18,481
Patient 35				13,302
Patient 36				16,489
Patient 37				15,111
Patient 38				12,477
Patient 39				18,105

Patient 40				14,511
Patient 41	41,947			11,455
Patient 42				16,636
Patient 43				17,299
Patient 44				10,243
Patient 45	43,881			18,225
Patient 46	37,18			17,211
Patient 47	38,814	4,394	13,048	10,93
Patient 48	40,664			19,369
Patient 49	27,034			16,83
Patient 50	55,436			11,418
Patient 51	45,75	6,032	15,983	12,896
Patient 52	56,911	5,498	16,194	10,17
Patient 53	58,321	3,832	12,519	11,778
Patient 54	61,017	3,612	14,418	12,707
Patient 55	54,266	5,475	14,376	12,354
Patient 56	44,819	6,795	19,224	8,994
Patient 57	45,134	6,517	11,836	15,522
Patient 58	48,784	6,543	14,784	16,222
Patient 59				15,305
Patient 60	53,425	6,979	12,212	14,388
Patient 61	34,029	4,984	10,377	15,694
Patient 62	39,782	4,883	11,845	10,408

Table S13. Electrophysiological parameters.

	Low-voltage (%)	Conduction Block (%)	Conduction block and delay (%)	Length Conduction block and delay	Conduction velocity
Patient 1	0,709	3,012	4,217	16,000	0,919
Patient 2	1,489	0,241	2,530		0,896
Patient 3	0,000	0,000	0,000		1,054
Patient 4	0,355	1,506	4,317	16,000	0,916
Patient 5	46,992	0,000	0,000		0,512
Patient 6	1,066	0,121	4,232	4,000	0,908
Patient 7	1,729	1,732	3,313	16,000	0,966
Patient 8	7,692	5,832	9,113	32,000	0,787
Patient 9	10,478	0,000	0,602		0,878
Patient 10	1,867	1,515	3,030	12,000	0,913
Patient 11	2,933	2,121	5,833	20,000	0,916
Patient 12	2,553	1,205	6,145	20,000	0,908
Patient 13	2,432	0,313	1,719	10,000	0,914
Patient 14	0,213	0,000	2,169		0,867
Patient 15	0,269	0,000	0,154		1,007
Patient 16	5,323	10,870	18,261	14,000	
Patient 17	8,708	0,000	6,522		
Patient 18	11,576	0,585	11,550		
Patient 19	1,068	0,000	0,806		1,014

Patient 20	0,478	6,359	13,591	26,000	
Patient 21	1,872	0,000	1,818		0,925
Patient 22	1,997	1,058	4,409	10,000	0,901
Patient 23	4,521	7,831	15,813	32,000	0,724
Patient 24	0,213	0,361	3,735	8,000	0,791
Patient 25	1,991	0,127	2,038	6,000	0,865
Patient 26	1,609	0,307	1,074		1,000
Patient 27	18,767	1,832	8,855	34,000	0,896
Patient 28	0,134	0,000	0,000		0,991
Patient 29	0,638	0,602	5,542	4,000	0,780
Patient 30	0,213	1,816	3,753	10,000	0,911
Patient 31	0,214	0,000	0,122		1,133
Patient 32	0,710	0,806	3,625	12,000	1,003
Patient 33	0,355	0,201	2,811	8,000	1,002
Patient 34	6,809	0,000	5,663		0,769
Patient 35	13,220	12,954	18,281	62,000	0,790

Authors note:
Patient numbers do not correspond across all tables. Arbitrary orders of patient number were applied for each table.
